# Metabolic dialogues: regulators of chimeric antigen receptor T cell function in the tumor microenvironment

**DOI:** 10.1002/1878-0261.13691

**Published:** 2024-06-22

**Authors:** Josquin Moraly, Taisuke Kondo, Mehdi Benzaoui, Justyn DuSold, Sohan Talluri, Marie C. Pouzolles, Christopher Chien, Valérie Dardalhon, Naomi Taylor

**Affiliations:** ^1^ Pediatric Oncology Branch, National Cancer Institute National Institutes of Health Bethesda MD USA; ^2^ Université Sorbonne Paris Cité Paris France; ^3^ Université de Montpellier, Institut de Génétique Moléculaire de Montpellier, CNRS Montpellier France

**Keywords:** anti‐tumor immunotherapy, chimeric antigen receptor, immunometabolism, nutrient transporters, T cells, tumor microenvironment

## Abstract

Tumor‐infiltrating lymphocytes (TILs) and chimeric antigen receptor (CAR) T cells have demonstrated remarkable success in the treatment of relapsed/refractory melanoma and hematological malignancies, respectively. These treatments have marked a pivotal shift in cancer management. However, as “living drugs,” their effectiveness is dependent on their ability to proliferate and persist in patients. Recent studies indicate that the mechanisms regulating these crucial functions, as well as the T cell's differentiation state, are conditioned by metabolic shifts and the distinct utilization of metabolic pathways. These metabolic shifts, conditioned by nutrient availability as well as cell surface expression of metabolite transporters, are coupled to signaling pathways and the epigenetic landscape of the cell, modulating transcriptional, translational, and post‐translational profiles. In this review, we discuss the processes underlying the metabolic remodeling of activated T cells, the impact of a tumor metabolic environment on T cell function, and potential metabolic‐based strategies to enhance T cell immunotherapy.

## Graphical abstract

T cells engineered to express chimeric antigen receptors (CAR) are dependent on the transport and utilization of nutrients from the extracellular space, fueling their anti‐tumor activity. Nonetheless, the tumor microenvironment is characterized by a dearth of several key nutrients and alterations in metabolic scavenging systems. It is therefore critical to equip CAR T cells to optimally function in these unique environments.

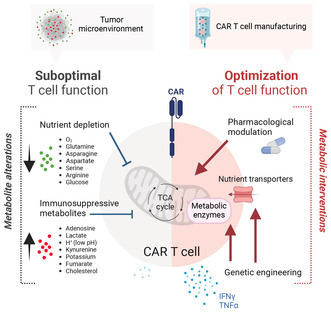

**Abbreviations**
2‐HG: 2‐hydroxyglutarate
A_2A_R: adenosine A2A receptor
ACAT1: acetyl‐CoA acetyltransferase
ADP: adenosine diphosphate
AHR: aryl hydrocarbon receptor
AKT: protein kinase B
AMP: adenosine monophosphate
AMPK: 5′ adenosine monophosphate‐activated protein kinase
APC: antigen presenting cell
ARG: arginase
ASCT2: alanine serine cysteine transporter 2
ASS: arginosuccinate synthase
ATP: adenosine triphosphate
BATF: basic leucine zipper transcription factor
BCMA: B‐cell maturation antigen
CAR: chimeric antigen receptor
CAT1: cationic amino transporter 1
CCR: C‐C chemokine receptor
CD: cluster of differentiation
cDC: conventional dendritic cell
CLL: chronic lymphocytic leukemia
CTLA4: cytotoxic T lymphocyte‐associated *protein* 4
CXCL: chemokine (C‐X‐C motif) ligand
CXCR: C‐X‐C motif chemokine receptor
CyTOF: cytometry by time of flight
eADO: extracellular adenosine
EGFR: epidermal growth factor receptor
eIF5A: elongation initiation factor 5A
ER: endoplasmic reticulum
ETC: electron transport chain
FAO: fatty acid oxidation
FH: fumarate hydratase
FOXP3: forkhead box P3
GAPDH: glyceraldehyde‐3‐phosphate dehydrogenase
GD2: disialoganglioside 2
GLS: glutaminase
GLUT: glucose transporter
GMP: *good manufacturing practices*
GOT1: glutamic‐oxaloacetic transaminase
GSK3: glycogen synthase kinase 3
HER2: human epidermal growth factor 2
HIF‐1α: hypoxia‐inducible factor 1α
IDH: isocitrate dehydrogenase
IDO: indoleamine‐pyrrole 2,3‐dioxygenase
IFN: interferon
IL: interleukin
IS: immune synapse
JmjC‐KDMs: jumonji C‐domain lysine demethylase
LCMV: lymphocytic choriomeningitis virus
LDH: lactate dehydrogenase
LXR: liver X receptor
MCT: monocarboxylate transporter
MDSC: myeloid‐derived suppressor cell
MGAT: mannose glycoprotein acetylglucosaminyltransferase
MHC: major histocompatibility complex
MPC: mitochondrial pyruvate carrier
mRNA: messenger RNA
mTOR: mammalian target of rapamycin
mTORC1: mammalian target of rapamycin complex 1
mTORC2: mammalian target of rapamycin complex 2
NFAT: nuclear factor of activated T‐cells
OE: overexpression
OTC: ornithine transcarbamylase
OXPHOS: oxidative phosphorylation
PD‐1: programmed cell death protein 1
PDH: pyruvate dehydrogenase
PD‐L1: programmed cell death ligand 1
PEP: phosphoenol pyruvate
PGC1α: pparg coactivator 1 alpha
PGE2: prostaglandin E2
PI3K: phosphoinositide 3‐kinase
PRODH2: proline dehydrogenase 2
RBD: receptor binding domain
ROS: reactive oxygen species
SAM: S‐Adenosyl methionine
SERCA: sarcoendoplasmic reticulum calcium ATPase
SLC: solute carrier
SNAT2: sodium‐dependent neutral amino acid transporter 2
TAM: tumor‐associated macrophage
TCA cycle: tricarboxylic acid cycle
TCR: T cell receptor
TDO: tryptophan 2,3‐dioxygenase
T_EFF_
: effector T cell
TET: ten‐eleven translocation
T_EX_
: exhausted T cell
Tf: transferrin
T_H_
: helper T cell
TME: tumor microenvironment
T_MEM_
: memory T cell
T_N_
: naïve T cell
TRC: tumor‐repopulating cells
T_REG_
: regulatory T cell
T_SCM_
: stem cell‐like memory T cell
VitC: vitamin C
ZAP‐70: Zeta‐chain‐associated protein kinase‐70
α‐KG: alpha‐ketoglutarate

## 1 Introduction

Our ability to optimally respond to pathogens, toxins, and foreign substances is largely controlled by the activation of T lymphocytes. T cells have also emerged as a potent tool in the treatment of cancer, providing robust anti‐tumor activity. However, the potential of a T cell to mount an immune response depends on an increase in its energetic state. Intracellular ATP is generated via the transport of a wide range of fuels from the extracellular space and these fuels are then catabolized through processes such as glycolysis, oxidative phosphorylation, and fatty acid oxidation, among others. Indeed, the energetic profile of the cell is intricately related to its ability to harness these resources, providing ATP for the cell [1, 2, 3, 4]. These fuels also provide biosynthetic intermediates supporting nucleic acid, amino acid, and phospholipid synthesis as well as the production of reducing equivalents to maintain the cell's redox state [1, 5, 6]. Moreover, metabolic pathway utilization governs the cell's epigenetic landscape, both directly, through the production of intermediates like acetyl‐CoA that contribute to histone acetylation, and indirectly, by generating metabolites that regulate enzymes such as demethylases [7, 8]. In this manner, fuel uptake and utilization govern the differentiation and function of a T lymphocyte, resulting in a wide range of immune responses. Here, we review the impact of metabolic pathways on T cell differentiation and focus on the role of metabolite shifts in the tumor microenvironment, conditioning the function and persistence of anti‐tumor T cells.

## 2 Differential utilization of metabolic pathways regulates T cell fate and anti‐tumor function

### 2.1 Dynamic metabolic remodeling conditions T cell activation and differentiation

#### 2.1.1 Metabolic regulation of naïve T cells

Naive T cells (T_N_) are continuously patrolling through the peripheral blood and secondary lymphoid tissues to achieve immune surveillance. The size of the T_N_ pool in the peripheral lymphoid compartment is tightly regulated and its maintenance is driven by homeostatic cytokines, in particular IL‐7, as well as T cell receptor (TCR)‐mediated tonic signals [9]. The metabolism of T_N_ is characterized by basal levels of ATP synthesis, necessary for the cytoskeletal remodeling associated with migration, and low levels of macromolecule biosynthesis that maintain cellular homeostasis. IL‐7 and low‐affinity TCR‐ major histocompatibility complex (MHC)‐peptide interactions maintain metabolic activity in T_N_, at least in part through glucose uptake via low levels of the GLUT1/SCL2A1 transporter [10, 11, 12, 13]. Overall, T_N_ metabolism relies mainly on mitochondrial oxidative phosphorylation (OXPHOS), fueled by glucose‐derived pyruvate as well as fatty acid oxidation. However, metabolic pathways in T_N_ can be quickly induced, due to the presence of a high level of untranslated mRNAs encoding metabolic enzymes and transporters as well as a rapid turnover of the transcription factors that regulate quiescence [14, 15]. Indeed, the high number of idling ribosomes in T_N_ provides the capacity to rapidly translate these mRNAs and remodel T cell metabolism within minutes following the engagement of TCR and CD28 costimulatory pathways, enabling a rapid switch to an effector program (T_EFF_). The activation of the PI3K‐AKT‐ mammalian target of rapamycin (mTOR) axis upregulates cell surface nutrient transporter expression and subsequent nutrient uptake. This leads to a massive increase in both aerobic glycolysis and OXPHOS, albeit with a bias towards the former [16, 17, 18, 19, 20]. Indeed, increased OXPHOS is required for the exit of T_N_ from quiescence and this process is regulated by mTORC1‐induced mitochondrial biogenesis [21, 22]. Upon TCR‐engagement, the mitochondrial mass polarizes at the immune synapse (IS), enabling efficient calcium buffering and local ATP production that sustain IS formation and migration [23, 24]. Moreover, mitochondrial reactive oxygen species (ROS) generated by the electron transport chain (ETC) complex III activate NFAT signaling and IL‐2 induction after antigen encounter, contributing to T cell activation [25]. The higher energetic state, resulting from a local increase of ATP production, sustains a massive increase in protein synthesis and a concurrent remodeling of the proteome through the non‐redundant activities of mTORC1, the MYC transcription factor, and translation initial factors [15, 26, 27, 28, 29]. Importantly though, there is an intricate crosstalk between OXPHOS and glycolysis. mTORC1/MYC signaling also increases glycolysis, promoting the production of biosynthetic intermediates that are critical for T cell proliferation and differentiation.

#### 2.1.2 Metabolic programs associated with T cell differentiation

Following activation of CD4^+^ T_N_, the recruitment of different cytokine‐induced signaling cascades and transcription networks results in the differentiation of T helper cells (T_H_) with distinct phenotypes and properties. Of note, the polarization of CD4^+^ T cells to a T_H_ effector versus suppressor fate is tightly coordinated with the establishment of different metabolic programs. T_H_1, T_H_2, and T_H_17 effector cells are highly glycolytic while suppressive regulatory T cells (T_REG_) rely mostly on lipid oxidation [30, 31]. In accord with the high level of IFNγ produced by T_H_1 effectors, regulated by the master T_H_1 transcription factor, Tbet, both Tbet and IFNγ expression are coupled to a glycolytic program [32, 33]. Glutaminolysis and the anaplerotic production of alpha‐ketoglutarate (αKG), feeding the tricarboxylic acid cycle (TCA), is also critical for T cell polarization. The absence of glutamine‐derived αKG inhibits T_H_1 polarization and biases cells towards a T_REG_ fate [34, 35]. Conversely, high levels of exogenous αKG inhibit T_REG_ polarization, resulting in an inflammatory T_H_1‐like phenotype [36]. Nonetheless, the implication of glutaminolysis in T cell differentiation is complex as glutaminase‐1 (GLS1)‐deficient CD4^+^ T cells initially exhibit elevated T_H_1 function but these cells become exhausted over time [37].

These data highlight the importance of metabolic shifts in controlling the balance between T_H_1 and T_REG_ differentiation, but it is also important to note that metabolic programs regulate the lineage fate of T_H_17 cells as compared to T_REG_. *De novo* fatty acid synthesis is required for T_H_17 while T_REG_ acquire the ability to take up exogenous fatty acids that are used in fatty acid oxidation (FAO) [38]. T_H_17 and T_REG_ also diverge with regards to their utilization of other nutrients and their dependence on metabolic programs. Specifically, T_REG_ cells can flourish in low glucose environments and indeed, expression of the T_REG_ transcription factor Foxp3 inhibits glycolysis [39]. In contrast, the hypoxia‐inducible factor HIF1α promotes T_H_17 differentiation over T_REG_, at least in part via an enhanced glycolytic program mediated by the upregulation of GLUT1 and other glycolytic enzymes [40, 41]. Arginine‐based polyamine metabolism varies between T_H_17 and T_REG_. T_H_17 cells are characterized by significantly higher levels of polyamines than T_REG_ cells. Furthermore, inhibition of polyamine generation, leading to a remodeling of the transcriptome and epigenome, skews pathogenic T_H_17 cells to a T_REG_‐like fate [42, 43]. Higher levels of glutamate transamination also occur in T_H_17 compared to T_REG_, leading to the accumulation of αKG and 2‐hydroxyglutarate (2‐HG) as well as methylation of the Foxp3 locus. In line with these observations, inhibition of the transaminase GOT1 is associated with a block in T_H_17 differentiation and the reprogramming of these cells to a T_REG_ fate [44]. Thus, the crosstalk between metabolic pathways, epigenetic modifications, and transcription factor expression plays a crucial role in regulating Th cell plasticity.

#### 2.1.3 Metabolic specificities of memory T cells

When compared to T_EFF_ cells, memory T cells (T_MEM_) display a more quiescent but metabolically primed metabolism, dependent on fatty acid oxidation, OXPHOS, and autophagy [45]. Mitochondrial remodeling participates in this process. The acquisition of elongated‐fused mitochondria with a tight cristae organization is associated with a higher mitochondrial reserve capacity and optimized efficiency of the ETC, creating a bioenergetic advantage for rapid recall [46, 47, 48]. Also, T_MEM_ maintains a higher level of translational activity compared to T_N_ cells with a two fold higher turnover of ribosomal and glycolytic enzymes, promoting a state of readiness that optimizes the recall response [15]. This metabolic divergence between memory and effector subsets is established within the early phases of T cell activation through an asymmetric sorting of the transcription factor MYC and amino acid transporters – before the first cell division. T cells with high MYC/high levels of transporters are capable of mounting a robust primary response while conversely, cells with low MYC/low levels of transporters support the generation of a memory response [49, 50]. Importantly, mTORC1 activity is also asymmetrically inherited after the first cell division through a mechanism notably dependent on amino acid influx. Notably, increasing this metabolism‐coupled asymmetric cell division in aged mice restores their potential to develop memory T cells. Thus, metabolic remodeling occurs throughout all stages of CD4^+^/CD8^+^ T cell activation and differentiation.

#### 2.1.4 Metabolic rewiring in exhausted T cells

Finally, the acquisition of an exhausted T cell phenotype is linked to metabolic rewiring. Importantly, repeat stimulations of the TCR within a given T cell, such as that occurring in the context of a chronic viral infection or tumor, lead to a dysfunctional T cell state commonly referred to as exhaustion [51, 52, 53]. In these contexts of chronic stimulation, T cells upregulate inhibitory molecules such as PD‐1 and CTLA‐4, and their engagement, either on anti‐tumor T cells or T_REG_, results in changes in FAO and glycolysis [54, 55, 56, 57]. Moreover, high levels of lactic acid in glycolytic tumors result in the upregulation of PD‐1 on T_REG_ [55, 58, 59, 60]. Furthermore, mitochondrial biogenesis is negatively impacted through a reduction in the expression of the transcriptional coactivator PGC1α [58, 61]. Indeed, mitochondrial dysfunction is a hallmark of TILs; Intra‐tumoral T cells display a reduction in mitophagy and accumulate depolarized mitochondria and mitochondrial ROS, thereby accelerating their differentiation through a terminally exhausted phenotype [62, 63, 64, 65]. Thus, overexpression of PGC1α may represent a therapeutic strategy via which CAR T cell exhaustion can be attenuated [61]. More generally, modulating the expression of candidate genes that impact metabolic pathways presents an area of research that holds much promise for improving the fitness of CAR T cells. Some of the genes that are being targeted in CAR T cells are presented in Section 5.2.

### 2.2 Crosstalk between T cell metabolic pathways and function: Signaling, epigenetic modifications, and post‐transcriptional regulation

**Fig. 1:**
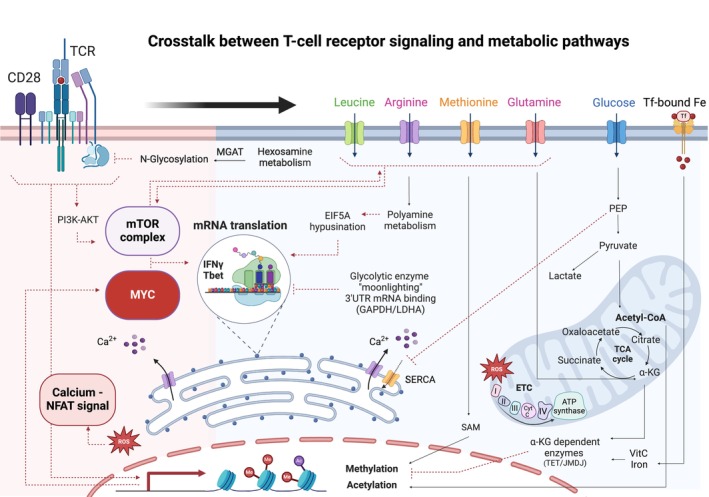
Crosstalk between T cell receptor signaling and metabolic pathways. TCR/CD28 signaling induces the expression of SLC transporters that regulate the entry of a large array of metabolites—glucose, glutamine, leucine, arginine, methionine, and iron, among others—enabling a massive induction of downstream pathways. The crosstalk between TCR signaling and intracellular metabolites modulates multiple critical cellular processes including, but not limited to, mTOR signaling, calcium/NFAT signaling, protein translation, N‐glycosylation, and epigenome remodeling. Ac, acetyl group; AKT, protein kinase C; ATP, adenosine triphosphate; Ca^2+^, calcium; CD28, cluster of differentiation 28; CytC, cytochrome C; EIF5A, elongation initiation factor 5A; ETC electron transport chain; Fe, iron; GAPDH, glyceraldehyde‐3‐phosphate dehydrogenase; IFNγ, interferon gamma; JMDJ, jumonji C‐domain lysine demethylases; LDHA, lactate dehydrogenase A; Me, methyl group; MGAT, mannose glycoprotein acetylglucosaminyltransferase; mTOR, mammalian target of rapamycin; NFAT, nuclear factor of activated T cells; PEP, phosphoenol pyruvate; PI3K, phosphoinositide 3‐kinase; ROS, reactive oxygen species; SAM, S‐adenosyl methionine; SERCA, sarcoendoplasmic reticulum calcium ATPase; SLC, solute carrier; TCA, tricarboxylic acid; TCR, T cell receptor; TET, ten‐eleven translocation; Tf, transferrin; VitC, vitamin C; α‐KG, alpha‐ketoglutarate.

Over the last decade, it has been increasingly recognized that T cell metabolism is not only a downstream event of TCR and cytokine signaling pathways but also impacts T cell function, at levels beyond energy production and building blocks for biosynthesis. Indeed, increases in intracellular metabolites, via nutrient transporter upregulation as well as *de novo* biosynthesis, regulate signaling changes that are intricately connected to metabolic programs that differ across T cell subsets (Fig. 1). One critical pathway that integrates extracellular signals with the metabolic environment of the cell is mTOR, a serine–threonine kinase. mTOR signaling is mediated via mTORC1 and mTORC2 complexes, tightly regulated by the intracellular concentration of amino acids. mTORC1 activity is activated by a multi‐step approach. Firstly, amino acids inhibit negative regulators of mTOR, including Sestrins, CASTOR, and SAMTOR. Secondly, amino acids activate mTORC1 on lysosomes via Rag and Rheb GTPases [66, 67, 68, 69]. mTORC2 is also activated by growth factors but the specific pathways are less well‐defined [70]. mTORC1 activity is required for both T_H_1 and T_H_17 differentiation while mTORC2 activity is a prerequisite for T_H_2 differentiation [4, 71, 72]. Furthermore, mTORC1 activation inhibits T_REG_ differentiation and function [73, 74, 75, 76, 77, 78]. Consistent with the critical role of glucose and amino acids in positively regulating mTORC1 activity, conditions that limit these metabolite levels block effector T cell generation but not T_REG_ differentiation [35, 79, 80, 81, 82].

T cell fate is modulated by both “top‐down” and “bottom‐up” metabolic signaling cascades, as proposed by Shyer et al. [83] TCR and extracellular signals are responsible for metabolic adaptations within the cell but metabolic changes also govern the potential of the T cell to respond to these engaged signaling networks. Moreover, metabolic signals act directly at the level of the TCR itself. MGAT5, an enzyme in the *N*‐glycosylation pathway, restricts TCR recruitment while conversely, its absence decreases the TCR activating threshold resulting in autoimmunity [84, 85]. In the context of “bottom‐up” signaling, metabolic intermediates and enzymes play roles outside of their canonical metabolic pathways. Indeed, Ho *et al*. elegantly showed that TCR‐induced increases in the glycolytic intermediate phosphoenolpyruvate (PEP) are not just important for glycolysis. PEP also sustains calcium release from the endoplasmic reticulum by directly blocking SERCA ATPase, thereby promoting NFAT signaling [86].

With regards to enzymes involved in glycolysis, glyceraldehyde 3‐phosphate dehydrogenase (GAPDH) and lactate dehydrogenase A (LDHA) exhibit important moonlighting activities in T cells, binding to the 3′ UTR mRNA of IFNγ and repressing its translation. The repressive RNA‐binding protein activity of GAPDH and LDHA is released when these enzymes are engaged in glycolysis, promoting the translation of IFNγ in a manner that couples glycolytic activity with cytokine release [32, 87]. Moreover, the polyamine‐derived amino acid hypusine, whose generation is generally dependent on arginine catabolism, regulates key effector functions in T cells at the translational level. Hypusination of eIF5A promotes translation of the transcription factor Tbet as well as IFNγ and IFNα among others [27, 88, 89, 90].

Metabolic fluxes also play a critical role in the epigenetic landscape of T cells. Acetyl‐CoA metabolism influences histone acetylation through the activity of histone acetyltransferases [7]. Reduction of glucose‐derived acetyl‐CoA by disruption of GLUT3‐mediated glucose uptake or pyruvate dehydrogenase (PDH) decreases histone acetylation, thereby attenuating the T_H_17‐associated transcriptional program [91, 92]. Conversely, the attenuation of mitochondrial pyruvate carrier 1 (MPC1) activity leads to an inhibition of pyruvate entry into the mitochondria. This results in an increased intracellular pool of acetyl‐CoA and subsequent histone acetylation, driving differentiation to a memory‐like phenotype [93]. Moreover, histone methylation, requiring the transfer of 3 methyl groups onto arginine and lysine histone residues, plays a critical role in T cell fate [94, 95]. The main source of these methyl groups is S‐adenosylmethionine (SAM), derived from methionine and one‐carbon metabolism. In this regard, it is interesting to note that the high expression of the methionine transporter SLC43A2 in certain tumors reduces methionine availability for T cells, thereby attenuating T cell cytotoxicity [96].

Loss of a mitochondrial enzyme, isocitrate dehydrogenase 2 (IDH2), results in a decrease in the reductive carboxylation of glutamine, which in turn attenuates the activity of the KDM5 histone demethylase and promotes T cell memory formation [97]. Indeed, Jumonji C‐domain lysine demethylases (JmjC‐KDMs), ten‐eleven translocation (TET) DNA cytosine‐oxidizing enzymes, and prolyl hydroxylases (PHDs) are all regulated by a large array of metabolism‐related factors – including α‐ketoglutarate and succinate TCA cycle metabolites, 2‐HG, iron, ascorbate, and oxygen levels – underscoring the intricate regulation of T cell fate by its metabolic environment [98]. This multi‐level crosstalk, together with the differential utilization of metabolic pathways in T cell subsets, highlights the large potential for metabolic interventions to improve T cell immunotherapy. However, it also exposes the potential complexity of these types of strategies.

## 3 Nutrient uptake through metabolite transporters governs the metabolic fitness of T lymphocytes

Metabolite transporters mediate the uptake of key nutrients including amino acids, sugars, nucleosides, iron, and fatty acids, thereby playing a critical role at the interface of the metabolic environment and intracellular metabolic pathways (Fig. 1). In the context of cytotoxic T cells, proteomic studies have shown that the most quantitatively prominent proteins in cytotoxic T cells include metabolic regulators, with at least 72 metabolite transporters [99, 100]. These transporters belong to the SLC (solute carrier) superfamily of multi‐membrane spanning proteins, containing more than 440 recognized members that mediate the intracellular entry of a large number of substrates, with variable levels of specificity [100]. Importantly though, the range and density of metabolite transporters that are expressed on T cells vary substantially as a function of the cell's activation state [99].

The study of metabolite transporters has been restricted by the lack of reliable antibodies against their extracellular domains, likely because of their low immunogenicity and their high conservation during evolution [101]. An innovative tool set has been developed to overcome this limitation, taking advantage of the discovery that gamma‐like envelopes of retroviruses and endogenous retroviral sequences use metabolite transporters as receptors, [12, 101, 102, 103, 104]. Using receptor binding domain (RBD) fusion proteins derived from retroviral envelopes, the cell surface expression of GLUT1/SLC2A1 and ASCT2/SLC1A5 glucose and glutamine transporters, respectively, have been shown to be rapidly increased at the surface of CD4^+^ T cells, within 2 h of TCR stimulation [19]. Furthermore, these tools have revealed the potential of T cells to engage compensatory mechanisms. Both GLUT1 and ASCT2 surface expression are significantly upregulated in response to glucose deprivation on naïve T cells, potentially explaining recent data showing that transient glucose deprivation can prime T cells and increase their anti‐tumor activity following adoptive transfer into tumor‐bearing mice [19, 105].

As indicated above, nutrient uptake in T lymphocytes is mediated by several transporters with a certain level of redundancy. Nonetheless, it is important to note that the deletion of a single transporter can lead to important functional consequences and these consequences can differ between T cell subsets. Ablation of the glucose transporter Glut1 in murine T cells has been found to attenuate CD4^+^ effector T cell function as well as CD4^+^‐mediated inflammatory responses. However, it does not appear to be required for CD8^+^ or T_REG_ function, likely due to a compensatory activity of the Glut3 glucose transporter [79]. Indeed, Glut3 ablation in murine T cells results in a different phenotype, negatively impacting T_H_17 effector function and T_H_17‐mediated autoimmunity through a reduction in glucose‐derived acetyl‐CoA and disturbed epigenetic regulation of inflammatory genes [92]. Moreover, deficiency of the glutamine transporter Asct2/Slc5a1 impairs T_H_1 and T_H_17 differentiation and decreases the generation of CD4^+^ memory populations in older mice, but notably, T_REG_ generation and function are not impacted [82]. Consistent with these data, glutamine deprivation significantly attenuates T_H_1 but not T_REG_ differentiation [34, 35]. In contrast, inhibition of the large neutral amino acid transporter Slc7a5/Lat1 has a more marked effect. Namely, it abrogates antigen‐induced effector T cell proliferation and function, most likely due to the loss of leucine‐mediated mTORC1 complex activation and Myc expression [81].

These examples highlight the importance of metabolite transporters in T cell effector generation and responsiveness to antigens. Nonetheless, recent work has unveiled the potential for transporter downregulation to improve the potential for T cell differentiation and function. An *in vivo* CRISPR screen, performed in the context of lymphocytic choriomeningitis mammarenavirus (LCMV) infection in mice, found that the Slc7a1/Cat1 cationic amino acid transporter and the Slc38a2/Snat2 neutral amino acid transporter both negatively regulate CD8^+^ memory T cell generation. Indeed, deletion of either of these transporters modulates mTORC1 signaling, improving the persistence and *in vivo* killing capacity of CD8^+^ memory T cells [106]. Moreover, deletion of the lactate transporter Slc16a1/Mct1 in T_REG_ attenuates their function, thereby decreasing tumor growth and enhancing the responsiveness of effector T cells to immune checkpoint blockade [107]. Finally, strategies that increase surface levels of metabolite transporters on cytotoxic T cells point to complex and intricate connections that result in the fine‐tuning of effector functions. For example, high levels of GLUT1 expression are associated with enhanced IFNγ and IL‐17 production but may decrease long‐term persistence of T cells engineered to express an anti‐tumor chimeric antigen receptor (CAR) [108]. Together, these studies show that multiple metabolite transporters serve as key drivers of T cell metabolic fitness, making them promising targets in the immunotherapy arsenal.

## 4 Immunosuppressive metabolic environments

**Fig. 2:**
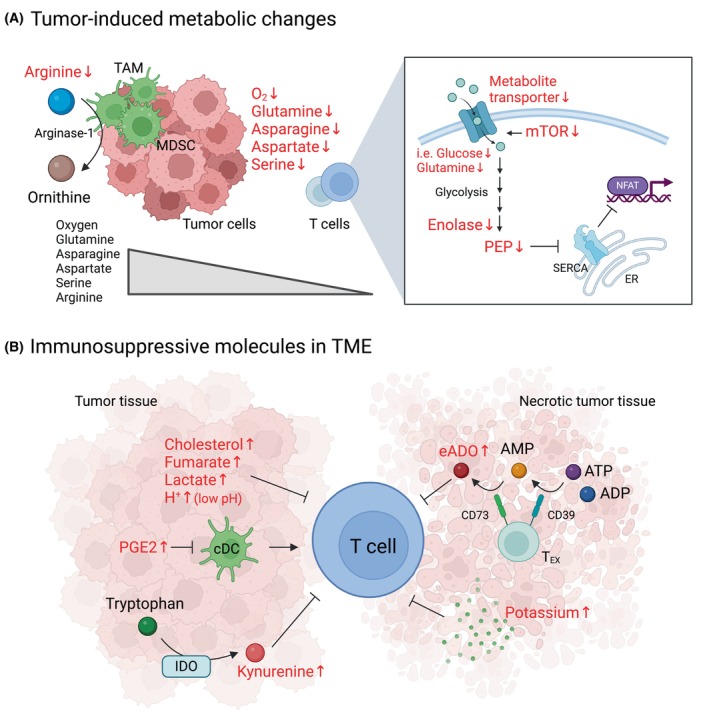
An immune hostile tumor microenvironment: Impact on T cell function. (A) The high uptake of diverse nutrients by tumor cells frequently results in the selective depletion of glutamine, asparagine, aspartate, serine, and arginine within the TME, leading to their decreased availability for anti‐tumor T lymphocytes. Moreover, arginase‐1, produced by tumor‐infiltrating TAM and MDSC, catabolizes arginine to ornithine, further limiting the utilization of arginine by tumor‐infiltrating cytotoxic T cells. The TME is often further compromised by suboptimal gas exchange, resulting in low oxygen concentrations (left). A limited accessibility to nutrients results in decreased mTOR signaling in tumor‐infiltrating T cells which in turn results in lower levels of cell surface metabolite transporters such as GLUT1. Decreases in glycolytic enzymes such as enolase result in the reduced generation of PEP, attenuating Ca^2+^‐NFAT activity via SERCA activity (right). (B) An abundance of tumor‐related metabolites suppresses T cell function. These include cholesterol, fumarate, lactate, low pH (secondary to high lactate secretion by tumor cells), and kynurenine – converted from tryptophan by IDO. PGE2 inhibits cDC function, thereby impairing T cell immune responses. Furthermore, ATP and ADP, released from dying cells, are converted to AMP and eADO by CD39 and CD73, respectively, on the surface of exhausted T cells. eADO as well as potassium from necrotic tumor tissue suppress T cell activity in the TME. ADO, aldehyde deformylating oxygenase; ADP, adenosine diphosphate; AMP, adenosine monophosphate; ATP, adenosine triphosphage; CD39, cluster of differentiation 39; CD73, cluster of differentiation 73; cDC, conventional dendritic cells; eADO, extracellular adenosine; ER, endoplasmic reticulum; GLUT1, glucose transporter; IDO, indoleamine 2, 3‐dioxygenase; MDSC, myeloid‐derived suppressor cell; mTOR, mammalian target of rapamycin; NFAT, nuclear factor of activated T cells; PEP, phosphoenolpyruvate; PGE2, prostaglandin E2; SERCA, sarcoendoplasmic reticulum calcium ATPase; TAM, tumor‐associated macrophage; TME, tumor microenvironment.

The tumor microenvironment (TME) is characterized by distinctive metabolic features such as changes in the concentrations of specific nutrients and adjustments in metabolic scavenging systems. Within necrotic tumor core tissue, poorly vascularized regions, and areas of high tumor growth, T cells compete for limited oxygen and nutrient supplies [109]. Moreover, the metabolic parameters of the TME display a high degree of heterogeneity and compartmentalization, increasing the complexity for immune cells to adapt within these different metabolic niches. Globally though, the TME presents a modified metabolic environment that results in compromised immunosurveillance [110]. As discussed in Section 2, an effective anti‐tumor T cell response is dependent on the T cell's ability to increase nutrient uptake but within a tumor, the conditions resulting in nutrient deprivation are likely to disrupt effector T cell differentiation and function. Indeed, T cells isolated from patients with renal cell carcinoma as well as chronic lymphocytic leukemia (CLL) were found to exhibit suboptimal metabolic profiles with attenuated glycolytic activity and mitochondrial dysregulation [111, 112, 113]. This metabolic dysfunction is intimately associated with T cell exhaustion and impairment of memory formation, both of which represent significant barriers to successful T cell immunotherapy (Fig. 2).

Several murine models have been harnessed to dissect the mechanisms via which the competition for nutrient resources impacts anti‐tumor immunity. Many studies have focused on glucose availability and indeed, in a mouse sarcoma model, limited glucose resources has been found to be a major factor associated with impaired tumor‐infiltrating T cell responses [54]. Glucose availability can also be limited by non‐tumor cells. In a murine colorectal cancer model, tumor‐infiltrating myeloid cells were found to consume the highest amounts of glucose, potentially limiting glucose uptake by T cells [114]. In this regard, it is interesting to note that strategies that increase glucose uptake by T cells may have clinical relevance. Overexpression of Glut1 was found to restore glycolytic activity in T cells in a murine leukemia model [113]. Moreover, checkpoint blockade antibodies against CTLA‐4, PD‐1, and PD‐L1 appear to improve T cell cytotoxicity and IFNγ production, at least in part through increases in glycolysis in syngeneic leukemia and solid tumor models [54].

As described in Section 2.2, glycolysis‐derived metabolites and enzymes play a crucial role in anti‐tumor T cell cytotoxicity. Notably though, the impaired glycolytic activity in tumor‐infiltrating T cells is not necessarily the result of low glucose availability but rather, it can also be related to intrinsic alterations in the T cell's metabolic machinery. For example, decreased levels of the PEP glycolytic intermediate, required for calcium/NFAT signaling (Fig. 1), can be due to glucose deprivation or to a reduced activity of enolase 1, the rate‐limiting step in PEP production [86]. Indeed, tumor‐infiltrating T cells in a mouse model of melanoma exhibited attenuated enolase 1 activity, indicating yet another glycolytic step that can be negatively impacted in the tumor microenvironment [115].

Beyond the critical role of glucose, decreased amino acid availability has been shown to significantly impair anti‐tumor responses. In this regard, it is important to note that several critical amino acids are highly depleted within the TME of both murine and human tumors. These include glutamine, arginine, aspartate, asparagine, and serine [116, 117, 118, 119]. As described in Section 2, these amino acids contribute to optimal T cell signaling, translation, and epigenetic remodeling (Figs 1 and 2A) [35, 120, 121, 122, 123, 124]. The finding that pharmacological inhibition of glutamine transport in a mouse triple‐negative breast cancer model increases the effector function of tumor‐infiltrating T cells [125] strongly supports the hypothesis that the tumor cell‐T cell competition for glutamine in the TME negatively impacts the latter. Arginine also directly impacts T cell function within the TME [109, 120, 121]. The high levels of arginine consumption by cancer cells together with the expression of arginine‐metabolizing enzymes [arginase (ARG)] by tumor‐associated macrophages (TAMs) and myeloid‐derived suppressor cells (MDSCs) contribute to the formation of an arginine‐depleted hostile environment [126]. Indeed, high levels of ARG1/2 within the TME have been correlated with poor prognosis in patients with ovarian carcinoma, pancreatic cancer, and head and neck squamous cell carcinoma [120, 127, 128, 129].

The immunosuppressive nature of the TME is also mediated by the secretion of metabolites (Fig. 2B). The high consumption of glucose by tumor cells results in the secretion of large amounts of lactate, blunting NFAT signaling in T cells [130] and skewing their differentiation. Furthermore, unlike conventional T cells, intra‐tumoral Foxp3^+^ T_REG_ cells display a metabolic program that is compatible with proliferation and sustained immunosuppressive activity that is sustained in glucose‐low/ lactate‐rich tumor environments [31, 105]. Conversely, inhibition of lactate dehydrogenase (LDH), coupled with IL‐21, may promote T cell stemness [131], but this may depend on the model system. For example, sodium lactate itself appears to promote T cell stemness in a murine colorectal model [132] and lactate oxidation sustains the anti‐tumor activity of CD8^+^ T cells in a murine melanoma TME [93].

Kynurenine and its metabolites are generated from tryptophan by cells in the TME expressing Indoleamine 2,3‐dioxygenase (IDO); these include tumor cells, TAMs, and MDSCs. Mechanistically, kynurenine, 3‐hydroxykynurenine, and 3‐hydroxyanthranilic acid can suppress T cell proliferation [133, 134]. Furthermore, the interaction of kynurenine and derived metabolites with the aryl hydrocarbon receptor (AHR) results in T_REG_ generation and tolerogenic myeloid cell differentiation [135]. Signaling through the AHR pathway is selectively active in IDO/TDO‐overexpressing tumors and is associated with resistance to immune checkpoint inhibitors [135]. There is also an extensive kynurenine‐mediated crosstalk between tumor‐repopulating cells (TRCs) and tumor‐infiltrating CD8^+^ T cells. IFNγ, produced by activated CD8^+^ T cells, triggers kynurenine secretion by TRCs which then feeds back on the intra‐tumoral T cells, upregulating PD‐1 expression and inducing T cell dysfunction [136, 137].

The metabolic state of a tumor can also result in the abnormal accumulation of metabolites. Mutations in fumarate hydratase (FH) lead to hereditary as well as sporadic forms of cancer [138, 139] and these malignancies are characterized by fumarate accumulation. Notably, recent work has shown that in a murine melanoma model, intra‐tumoral fumarate suppresses tumor‐infiltrating CD8^+^ T cells via succination of the ZAP‐70 protein tyrosine kinase [140]. Similarly, mutations in isocitrate dehydrogenase 1 (IDH1), occurring in malignant gliomas, result in the production of R‐2‐hydroxyglutarate (R‐2HG). This oncometabolite impairs CD8^+^ T effector function via inhibition of the glycolytic enzyme LDH [141, 142]. Thus, tumor cells secrete metabolites that negatively affect anti‐tumor T cells via an extraordinarily broad range of mechanisms (Fig. 2B).

In addition to metabolites secreted by live tumor cells, the necrosis of tumor cells results in the liberation of immunosuppressive components. Extracellular potassium, released by necrotic tumor cells, has been identified as an inhibitory chemical element limiting T cell effector function. The ionic imbalance inhibits metabolic signaling through the AKT and mTOR kinases, suppressing T cell effector function. Mechanistically, high levels of extracellular potassium limit nutrient uptake into T cells, inducing autophagy and reducing histone acetylation at effector and exhaustion loci [143, 144, 145].

Non‐tumoral cells also secrete metabolites that participate in the creation of a hostile TME. One example is the production of itaconate [product of immune‐responsive gene 1 (Irg1)] by MDSCs. Itaconate uptake by CD8^+^ T cells inhibits the biosynthesis of aspartate and serine/glycine, leading to decreased proliferation, cytokine production, and cytotoxic activity [146]. In this regard, it is interesting to note that the knockdown of *Irg1* reduces melanoma growth, inhibits the immune‐suppressive activities of MDSCs, promotes anti‐tumor immunity of CD8^+^ T cells, and enhances the efficacy of anti‐PD‐1 treatment [146].

Furthermore, ATP, actively released in response to cellular stress, is another key component of the TME [147, 148]. The CD39 and CD73 ecto‐nucleotidases catabolize ATP into extracellular adenosine (eADO) [149] and in turn, eADO can inhibit anti‐tumor responses through its binding to purinergic receptors expressed on T cells [148, 150]. In line with these results, the absence of CD39 has been shown to be associated with stem cell memory (T_SCM_)‐like TILs which exhibit long‐term persistence in patients responding to TIL therapies [151]. Conversely, expression of CD39 on tumor‐infiltrating T cells is sufficient to limit anti‐tumor immunity [152], highlighting the important role of adenosine metabolism in regulating T cell function within the TME.

Oxygen tension is a critical factor modulating the TME. The high consumption of oxygen by tumor cells and the abnormal tumor blood vasculature generates hypoxic conditions that significantly impact T cell function. Furthermore, several studies have reported that the combination of intra‐tumoral hypoxic conditions and chronic antigen exposure provokes T cell exhaustion, with the hypoxic environment impairing responsiveness to checkpoint blockade [152, 153, 154].

While many nutrients and metabolic intermediates are depleted in the tumor microenvironment, this is not the case for lipid‐based metabolites. Cholesterol is notably enriched in tumor tissues [155] and its accumulation in tumor‐infiltrating CD8^+^ T cells results in the upregulation of T cell exhaustion markers and an ER‐stress‐XBP1‐dependent inhibition of anti‐tumor function [156]. Acyl‐coenzyme A:cholesterol acyltransferase1 (ACAT1)‐driven cholesterol esterification inhibits TCR signaling by binding to the transmembrane region of the TCRβ chain, disrupting TCR clustering. Interestingly, it appears that this pathway can be targeted for therapy. For instance, ACAT1 inhibition by pharmacological and gene‐editing approaches potentiates the anti‐tumor activity of T cells [157]. Furthermore, cholesterol‐driven liver X receptor (LXR) activation can result in differential effects as a function of the targeted T cell subset. Its activation has been found to inhibit IL‐9‐producing cytotoxic CD8^+^ T cell differentiation and associated anti‐tumor cytotoxicity [158]. In this context, it is notable that pharmacological modulators of lipid metabolism are being evaluated as part of the arsenal of anti‐tumor T cell therapies [159].

Prostaglandin E2 (PGE2) has also been detected in the TME, impairing the survival and anti‐tumor function of both CD4^+^ and CD8^+^ T cells [160, 161]. Similarly to other metabolites that inhibit the function of conventional but not regulatory T cells, PGE2 induces FOXP3 expression and enhances suppressive T cell function [162]. PGE2 also plays an indirect role in regulating T cell function. PGE2 downregulates IRF8 expression in intra‐tumoral type 1 dendritic cells (DCs) and the subsequent DC dysfunction results in an impaired CD8^+^ T cell infiltration within the tumor [163].

In light of the myriad factors and conditions that suppress T cell function in the tumor microenvironment, it is remarkable that anti‐tumor T cells can sometimes succeed in controlling tumor growth. In the next section, recent approaches that facilitate the ability of anti‐tumor T cells to overcome the immune hostile TME are discussed.

## 5 Metabolic interventions to optimize adoptive T cell immunotherapy

Adoptive T cell therapy represents a cutting‐edge strategy, designed to treat cancer patients through the transfer of anti‐tumor T cells. T cells engineered to express a CAR against a tumor antigen have demonstrated remarkable success in the treatment of human hematological malignancies [164, 165, 166]. Nonetheless, despite this remarkable success, CAR T‐treated patients do relapse, at least in part due to poor *in vivo* persistence and function of transferred CAR T cells [167]. Furthermore, the efficiency of CAR T cell approaches in the treatment of solid tumors remains limited [168]. The lack of a memory T cell phenotype in the transferred T cells and a suboptimal metabolic fitness of the CAR T cells may account for their constrained function, especially in the solid tumor microenvironment.

**Fig. 3:**
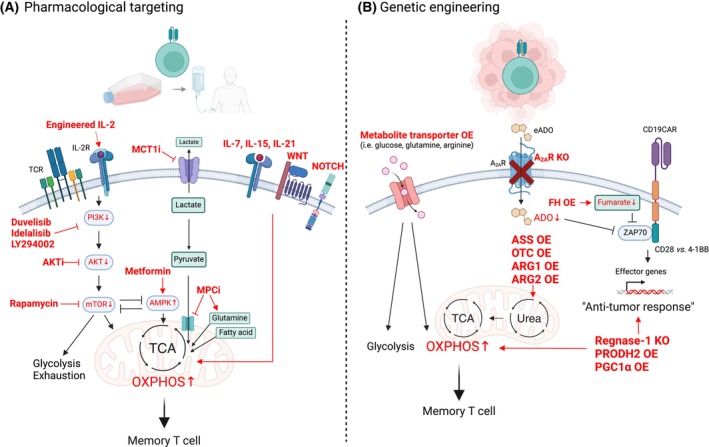
Potential metabolic interventions to optimize CAR T cell efficacy. (A) Diverse pharmacological manipulations have been employed during the *ex vivo* generation of CAR T cells in an attempt to improve their *in vivo* cytotoxicity and persistence. Engineered IL‐2, a partial agonist of the IL‐2 receptor, PI3K inhibitors (Duvelisib, Idelalisib, LY294002), AKT inhibitors, and rapamycin‐like molecules increase mitochondrial OXPHOS by modulating the PI3K‐AKT–mTOR signaling axis. Metformin stimulates AMPK, modulating mitochondrial OXPHOS; MPC inhibitors result in increased generation of acetyl‐CoA and histone acetylation with glutamine/fatty acid‐driven mitochondrial metabolism; and MCT‐1 inhibitors block lactate export, potentiating mitochondrial activity. IL‐7, IL‐15, IL‐21, WNT, and NOTCH‐mediated signal transductions also facilitate mitochondrial OXPHOS. Upregulation of mitochondrial reprogramming generally induces memory T cell formation and enhanced anti‐tumor activity. (B) Genetic manipulation of CAR T cells can be exploited to enhance anti‐tumor function. The type of costimulatory domain on the CAR construct will modulate its metabolic state. Regnase‐1 KO, PRODH2 OE, and PGC1α OE facilitate mitochondrial OXHOS and memory formation as well as increased transcription of effector function‐related genes. OE of metabolite transporters as well as enhanced expression of enzymes involved in their generation/catabolism, such as ASS, OTC, ARG1, and ARG2, may also stimulate anti‐tumor CAR T cell cytotoxicity. The harmful effects of tumor metabolites, such as fumarate and adenosine, in CAR T cells can be suppressed by overexpression of fumarate hydratase and knockdown of the adenosine A_2A_ receptor, respectively. A2AR, adenosine A2A receptor; ADO, adenosine; AKT, protein kinase B; AMPK, 5′ adenosine monophosphate‐activated protein kinase; ARG1‐2, arginase 1–2; ASS, arginosuccinate synthase; CAR, chimeric antigen receptor; CD19, cluster of differentiation 19; CD28, cluster of differentiation 28; eADO, extracellular adenosine; FH, fumarate hydratase; IL‐15, interleukin 15; IL‐2, interleukin 2; IL‐21, interleukin 21; IL‐2R, interleukin 2 receptor; IL‐7, interleukin 7; MCT‐1, monocarboxylate transporter 1; MCT1i, monocarboxylate transporter 1 inhibitor; MPC, mitochondrial pyruvate carrier; MPCi, mitochondrial pyruvate carrier inhibitor; mTOR, mammalian target of rapamycin; OE, overexpression; OTC, ornithine transcarboxylase; OXPHOS, oxidative phosphorylation; PGC1α, pparg coactivator 1 alpha; PI3K, phosphoinositide 3‐kinase; PRODH2, proline dehydrogenase 2; TCA, tricarboxylic acid; TCR, T cell receptor.

To overcome these limitations, much research has focused on the adoptive transfer of less differentiated memory T cells. Indeed, administration of T cells with a naïve and/or T_SCM_ phenotype has demonstrated robust anti‐tumor responses and long‐term remission in preclinical murine, macaque, and human models [169, 170, 171]. Importantly, mechanistic studies showing differences in the metabolism of T cell subsets (see Section 1) correlate with the distinct phenotypes of adoptively transferred CAR T cells in patients. The transcriptomic profiles of CAR T cells in complete‐responding patients with chronic lymphocytic leukemia (CLL) correlated with memory T cell formation and low glycolytic activity [172]. Indeed, pharmacological inhibition of glycolysis was found to result in the generation of increased frequencies of human central memory CAR T cells [172]. Interestingly, low glycolytic activity was previously found to enhance murine CD8^+^ T cell memory [173]. Thus, generating CAR T cells with a metabolic program that promotes an “early memory” T cell phenotype is likely to improve intra‐tumoral CAR T cell persistence and function, thereby promoting remission in patients (Fig. 3). Nonetheless, in the context of T cell‐based immunotherapies for solid tumors, it is critical that the anti‐tumor T cells have the potential to traffic and home to the tumor. Subsequently, they must navigate the tumor stroma network and mount an effective anti‐tumor response within the hostile tumor microenvironment (see Section 4). To this end, chemokine receptors and their ligands are attractive targets, modulating T cell recruitment/function and depleting MDSCs/TAMs [174]. Effector T cells may have an advantage as they express chemokine receptors such as CXCR3, that can promote T cell recruitment into solid tumors [175, 176]. Interestingly though, the upregulation of CXCR3 on anti‐tumor T cells within the tumor microenvironment may be even more important than its role outside the tumor. This upregulation appears to restore T cell responsiveness after anti‐PD1 checkpoint blockade by guiding intra‐tumoral CXCR3^+^ T cells to CXCL9‐producing CD103^+^ DCs in the context of several different tumors [177, 178]. More generally, much research has been invested in equipping CAR T cells with enhanced tumor targeting through the transgenic expression of chemokine receptors such as CXCR1/2, CCR4, CCR2b, and CXCR6 [174].

### 5.1 Pharmacological targeting of CAR T cells

To generate CAR T cells, T cells isolated from patients are stimulated through their TCR, transduced with a retroviral or lentiviral vector harboring the CAR transgene, and then are expanded *in vitro*. During this latter step, ectopic cytokines are added. While IL‐2 has been classically added, other cytokines such as IL‐7, IL‐15, and IL‐21 have also been found to support the generation of human CAR T cells [179, 180, 181]. Importantly, each cytokine differentially impacts T cell metabolism. Cytokines such as IL‐15 and IL‐21 have been reported to decrease glycolysis, increase fatty acid oxidation, and increase mitochondrial activity, thereby promoting the generation of CAR T cells that may present with superior metabolic fitness for *in vivo* persistence [182, 183, 184, 185]. This is also the goal behind the approach of rapidly generating patient CAR T cells following a rapid 24‐h stimulation with IL‐7 and IL‐15 alone [186]. Moreover, IL‐2 partial agonists were engineered in order to develop a molecule that allows T cell expansion but does not induce a metabolic program resulting in T cell exhaustion. One such molecule, H9T, promoted the generation of murine CAR T cells with an optimized mitochondrial fitness and these lymphocytes exhibited enhanced anti‐tumor efficacy in leukemia and melanoma models [187].

Pharmacological inhibitors can also be used to directly or indirectly target metabolic pathways. The utilization of duvelisib to inhibit PI3Kδ/γ during the manufacturing of CAR T cells from patients with CLL increased mitochondrial biomass, a metabolic change that was associated with an enhanced number of T_SCM_ and greater *in vivo* persistence and cytotoxicity [188]. Other PI3K inhibitors, such as idelalisib and LY294002, also favor early memory T cell generation and improve human CAR T cell function [189, 190]. The AKT and mTOR kinases are downstream of PI3K (Fig. 3A) and their pharmacological targeting by agents such as the AKT inhibitor VIII (AKTi), ipatasertib, and rapamycin have been found to result in similar effects in murine and human models [191, 192, 193]. The subsequent metabolic reprogramming modulates a FOXO1‐driven transcription program with upregulation of early memory T cell phenotypes, resulting in the induction of CXCR4 on human CAR T cells and increased infiltration into the bone marrow [192, 194]. In addition, other pathways have also been targeted such as activation of WNT using the GSK3β inhibitor TWS‐119 and activation of NOTCH via the DLL1 NOTCH ligand. Activation of both these pathways results in altered metabolic programs, and the hypothesis is that decreased glycolysis facilitates the generation of T_SCM_ and robust anti‐tumor activity [195, 196, 197, 198, 199]. Urolithin A (UA), an inducer of mitophagy that is a metabolite of pomegranate extract, was found to drive WNT signaling and promote the generation of murine T_SCM_ cells with high anti‐tumor cytotoxicity against a murine colorectal adenocarcinoma [200], highlighting the multiple interconnections between these pathways in T lymphocytes.

Another major research axis has focused specifically on modulating glucose availability as well as glycolysis, both during CAR T cell generation and in the final product [201]. Inhibition of the lactate transporter MCT1 results in a bias towards oxidative phosphorylation over glycolysis and while this intervention had no impact on the phenotype of human CD19 CAR T cells, their anti‐leukemia cytotoxicity was enhanced [202]. Furthermore, attenuation of pyruvate entry into the mitochondria, through inhibition of the SLC54A1/MPC1 transporter, increased the pool of acetyl‐CoA and associated histone acetylation. This led to a memory‐like phenotype of both murine and human CD19 CAR T cells with enhanced anti‐tumor activity [93]. The antidiabetic drug metformin – an inhibitor of mitochondrial respiratory chain complex I and activator of AMPK [203] – appears to promote memory T cell generation, improving the function of HER2 CAR T cells against human lung adenocarcinoma [204]. Moreover, the presence of metformin within the tumor microenvironment may be advantageous. The inclusion of a metformin‐containing hydrogel scaffold in human gastric carcinoma tissue improved CAR T cell infiltration and proliferation, likely by upregulating oxidative phosphorylation [205]. This summary highlights the large array of metabolic‐focused CAR T cell interventions that have been successfully tested in the lab (Fig. 3A). Therefore it will be of much interest to determine the potential efficacy of these interventions in the good manufacturing practices (GMP) manufacturing of CAR T cells for clinical trials.

### 5.2 Genetic manipulation of CAR T cells

The interventions described above are all dependent on extracellular cues. Notably, gene‐editing strategies, have been very successful, at least in preclinical models, by directly targeting metabolic pathways va overexpression or knockdown of specific genes. Indeed, overexpression of PGC1α, a transcription factor associated with mitochondrial biogenesis, rescues EGFR CAR T cells from mitochondrial dysfunction in the TME, improving their fitness and cytotoxicity against human lung cancer cells (Fig. 3B) [61, 206]. However, metabolic interventions are likely to differ as a function of the CAR target and the tumor model. Additionally, the impact of these interventions may vary as a function of the costimulatory domain incorporated into the CAR. CAR constructs generally include either a CD28 or 4‐1BB costimulatory domain and the downstream signaling moieties that are activated following engagement of CARs with these different domains are not equivalent [207, 208, 209, 210, 211]. Activation of distinct signaling cascades will, in turn, alter the cell's metabolic program, but interestingly, metabolic analyses comparing CD28 and 4‐1BB CARs have only been reported in non‐activated human T cells electroporated for transient CAR expression [212]. It will therefore be critical to review the role of the CAR costimulatory domain in driving CAR T cell metabolism in the context of the different therapeutic strategies discussed below.

The metabolism of CAR T cells can be directly altered by inhibiting or overexpressing metabolic enzymes and transporters. Arginine metabolism, discussed in Sections 2 and 3, plays a critical role in T cell proliferation and survival, and the TME is relatively depleted in arginine. To overcome arginine depletion, Mussai, De Santo and colleagues ectopically expressed enzymes involved in either arginine catabolism (ARG1/ARG2) or arginine resynthesis [arginosuccinate synthase (ASS) and ornithine transcarbamylase (OTC)] in CD33‐ as well as GD2‐targeted CAR T cells. They found that these modified CAR T cells exhibited enhanced *in vivo* anti‐tumor responses against human AML and neuroblastoma [213, 214]. Similarly, our group and others have modulated the expression of glucose, glutamine, and arginine transporters, among others, in order to enhance CAR T cell function in nutrient‐deprived tumor microenvironments (Fig. 3B) [108, 214].

Gene‐editing strategies have also been harnessed in an attempt to protect CAR T cells from the potentially harmful effects of tumor metabolites in the tumor microenvironment. To attenuate the negative effects of tumor‐generated fumarate (see Section 4), mouse and human CD19 CAR T cells were engineered to overexpress fumarate hydratase, thereby promoting their catabolism of intracellular fumarate. Notably, these CAR T cells demonstrated an enhanced anti‐tumor activity [140]. Several other groups took related approaches in an attempt to abrogate the negative effects of adenosine. CAR T cells engineered with a deletion in the adenosine receptor A_2A_R gene as well as CAR T cells overexpressing adenosine deaminase 1, catabolizing adenosine into inosine, exhibited greater anti‐tumor activity in several models, including mouse mammary cancer and human ovarian cancer.

CRISPR screening approaches in CD4^+^ as well as CD8^+^ T cells have been undertaken in an attempt to discover genes and gene networks that regulate T cell immunity in hematological malignancies as well as solid tumors [215, 216, 217, 218, 219, 220]. Given the importance of metabolism in T cell immunity, it is not surprising that many identified genes regulate metabolic pathways. One gain of function CRISPR screen identified proline dehydrogenase2 (Prodh2) as the top hit, increasing proline, as well as arginine metabolism. Most notably, PRODH2 overexpression in human CD22‐, BCMA‐, as well as HER2‐directed CAR T cells significantly increased anti‐tumor activity [217]. Another CRISPR screen found that deletion of Regnase 1, a native regulator of the basic leucine zipper ATF‐Like transcription factor (Batf), enhanced mitochondrial function as well as CAR T cell responsiveness [216]. Moreover, BATF itself has been found to promote T cell survival and anti‐tumor responsiveness of CAR T cells [221, 222, 223]. Nonetheless, the specific experimental conditions are critical because inverse effects have also been reported, knocking out BATF in human CAR T cells under exhaustion conditions was reported to improve cytotoxicity, and high BATF3 expression in TET2‐edited CAR T cells resulted in reduced cytotoxicity [224, 225]. While these discrepant results are likely due to underlying differences in T cell metabolism in leukemia and solid tumor models being evaluated, they hinder extrapolation to clinical trials in patients. Together, these data highlight the hurdles in advancing these targeted approaches to clinical trials but they also point to the extraordinary potential of harnessing metabolic pathways to promote anti‐tumor T cell responses.

To improve the therapeutic efficacy of CAR T cells, especially with regards to their application in the treatment of solid tumors, metabolic interventions by pharmacological and gene‐editing technologies present promising avenues. In this regard, it is notable that multidimensional omics data from both tumor and anti‐tumor immune cells can now be leveraged to identify novel metabolic targets [226]. Moreover, spatial organizations of metabolic programs have been revealed through the profiling of human tissues and cells at a single‐cell level using recently described techniques such as scMEP (single‐cell metabolic regulome profiling), SCENITH (Single Cell ENergetIc metabolism by profiling Translation inHibition), and scSpaMet (Single Cell Spatially resolved Metabolic), incorporating CyTOF, bulk metabolic assays, protein synthesis assays, and multiplex Imaging Mass Cytometry [227, 228, 229]. An understanding of the metabolic genes whose expression profiles are altered in tumors may promote the design of CAR T cells that are remodeled to better function within the metabolic complexity of TME [230, 231]. Indeed, single‐cell analyses of human hepatocellular carcinoma and human pancreatic ductal adenocarcinoma have recently revealed distinctive metabolic changes in methionine recycling, glutamine metabolism, and lipid accumulation [232, 233, 234, 235]. However, deducing the activity of a particular metabolic pathway from scRNAseq data can be challenging due to weak correlations between mRNA levels and protein levels. Additionally, there remains an inadequate understanding of the activity of metabolic enzymes and transporters within the highly interconnected metabolic networks [14, 15, 236]. System‐based computational modeling approaches that integrate mRNA levels of metabolic genes with the prior knowledge of these networks may help to overcome these limitations, promoting a more comprehensive view of single‐cell metabolic profiles [42, 236, 237]. The hope is that by improving the fitness of CAR T cells within tumor‐specific microenvironments, the outcomes of patients receiving CAR T cell therapy will be significantly improved.

## 6 Perspectives and conclusions: Mice and humans…

As highlighted 20 years ago in a review from Mestas and Hughes entitled “Of mice and not men: Differences between Mouse and Human Immunology,” 65 million years of evolution have resulted in significant differences between mice and humans. Genes impacting both innate and adaptive immunity differ in their expression profiles and functions, including critical T cell signaling molecules such as ZAP‐70 and the common gamma chain [238]. Moreover, some pathways are species‐specific. For example, while the mouse CD46 complement regulator does not contain any known signaling motifs, human CD46 is a critical regulator of macrophage and T cell function [239, 240]. These divergences are compounded by the particularities of mice strains and the controlled environments in which mice are maintained.

Mice are housed in specific pathogen‐free conditions resulting in the maintenance of a naïve immune environment, an anomalous state as compared to humans. The immune cell compartment of laboratory mice can be altered by exposing them to the microbiota of their wild counterparts – markedly modifying their responses to antigens [241, 242, 243, 244, 245] – but to date, this is not common in immune studies. Indeed, these disparities have led to conclusions regarding disease processes and treatments that are often not universally applicable, not just to humans but to other strains of mice and diverse conditions [246, 247, 248, 249]. Furthermore, while humanized mouse models are essential for preclinical studies of novel anti‐tumor receptors on human T cells within a human tumor setting, the absence of an adaptive immune system in these mice frequently complicates interpretation of the data. Nonetheless, while these examples highlight the myriad difficulties in extrapolating mouse experimental data into clinical trials for patients, syngeneic and humanized mouse models are powerful tools. Their optimization will be invaluable in the quest for safer and more efficient anti‐tumor immunotherapies.

## Conflict of interest

The authors declare no conflict of interest.

## Author contributions

JM, TK, VD, and NT conceived the manuscript and wrote the review. JM and TK designed the figs MB, JD, ST, MCP, and CC substantially contributed to the research and content of the review and edited the manuscript.

## References

[1] Almeida L , Lochner M , Berod L , Sparwasser T . Metabolic pathways in T cell activation and lineage differentiation. Semin Immunol. 2016;28(5):514–524. 27825556 10.1016/j.smim.2016.10.009

[2] Geltink RIK , Kyle RL , Pearce EL . Unraveling the complex interplay between T cell metabolism and function. Annu Rev Immunol. 2018;36:461–488. 29677474 10.1146/annurev-immunol-042617-053019 PMC6323527

[3] Makowski L , Chaib M , Rathmell JC . Immunometabolism: from basic mechanisms to translation. Immunol Rev. 2020;295(1):5–14. 32320073 10.1111/imr.12858 PMC8056251

[4] Yong CS , Abba Moussa D , Cretenet G , Kinet S , Dardalhon V , Taylor N . Metabolic orchestration of T lineage differentiation and function. FEBS Lett. 2017;591(19):3104–3118. 28901530 10.1002/1873-3468.12849

[5] MacIver NJ , Michalek RD , Rathmell JC . Metabolic regulation of T lymphocytes. Annu Rev Immunol. 2013;31:259–283. 23298210 10.1146/annurev-immunol-032712-095956 PMC3606674

[6] Lim SA , Su W , Chapman NM , Chi H . Lipid metabolism in T cell signaling and function. Nat Chem Biol. 2022;18(5):470–481. 35484263 10.1038/s41589-022-01017-3 PMC11103273

[7] Soriano‐Baguet L , Brenner D . Metabolism and epigenetics at the heart of T cell function. Trends Immunol. 2023;44(3):231–244. 36774330 10.1016/j.it.2023.01.002

[8] Yerinde C , Siegmund B , Glauben R , Weidinger C . Metabolic control of epigenetics and its role in CD8^+^ T cell differentiation and function. Front Immunol. 2019;10:2718. 31849941 10.3389/fimmu.2019.02718 PMC6901948

[9] Surh CD , Sprent J . Homeostasis of naive and memory T cells. Immunity. 2008;29(6):848–862. 19100699 10.1016/j.immuni.2008.11.002

[10] Barata JT , Silva A , Brandao JG , Nadler LM , Cardoso AA , Boussiotis VA . Activation of PI3K is indispensable for interleukin 7‐mediated viability, proliferation, glucose use, and growth of T cell acute lymphoblastic leukemia cells. J Exp Med. 2004;200(5):659–669. 15353558 10.1084/jem.20040789 PMC2212738

[11] Rathmell JC , Vander Heiden MG , Harris MH , Frauwirth KA , Thompson CB . In the absence of extrinsic signals, nutrient utilization by lymphocytes is insufficient to maintain either cell size or viability. Mol Cell. 2000;6(3):683–692. 11030347 10.1016/s1097-2765(00)00066-6

[12] Swainson L , Kinet S , Mongellaz C , Sourisseau M , Henriques T , Taylor N . IL‐7‐induced proliferation of recent thymic emigrants requires activation of the PI3K pathway. Blood. 2007;109(3):1034–1042. 17023582 10.1182/blood-2006-06-027912

[13] Wofford JA , Wieman HL , Jacobs SR , Zhao Y , Rathmell JC . IL‐7 promotes Glut1 trafficking and glucose uptake via STAT5‐mediated activation of Akt to support T‐cell survival. Blood. 2008;111(4):2101–2111. 18042802 10.1182/blood-2007-06-096297 PMC2234050

[14] Ricciardi S , Manfrini N , Alfieri R , Calamita P , Crosti MC , Gallo S , et al. The translational machinery of human CD4^+^ T cells is poised for activation and controls the switch from quiescence to metabolic remodeling. Cell Metab. 2018;28(6):895–906.e5. 30197303 10.1016/j.cmet.2018.08.009 PMC6773601

[15] Wolf T , Jin W , Zoppi G , Vogel IA , Akhmedov M , Bleck CKE , et al. Dynamics in protein translation sustaining T cell preparedness. Nat Immunol. 2020;21(8):927–937. 32632289 10.1038/s41590-020-0714-5 PMC7610365

[16] Ma EH , Verway MJ , Johnson RM , Roy DG , Steadman M , Hayes S , et al. Metabolic profiling using stable isotope tracing reveals distinct patterns of glucose utilization by physiologically activated CD8^+^ T cells. Immunity. 2019;51(5):856–870.e5. 31747582 10.1016/j.immuni.2019.09.003

[17] Levine LS , Hiam‐Galvez KJ , Marquez DM , Tenvooren I , Madden MZ , Contreras DC , et al. Single‐cell analysis by mass cytometry reveals metabolic states of early‐activated CD8^+^ T cells during the primary immune response. Immunity. 2021;54(4):829–844.e5. 33705706 10.1016/j.immuni.2021.02.018 PMC8046726

[18] Klein Geltink RI , O'Sullivan D , Corrado M , Bremser A , Buck MD , Buescher JM , et al. Mitochondrial priming by CD28. Cell. 2017;171(2):385–397.e11. 28919076 10.1016/j.cell.2017.08.018 PMC5637396

[19] Clerc I , Moussa DA , Vahlas Z , Tardito S , Oburoglu L , Hope TJ , et al. Entry of glucose‐ and glutamine‐derived carbons into the citric acid cycle supports early steps of HIV‐1 infection in CD4 T cells. Nat Metab. 2019;1(7):717–730. 32373781 10.1038/s42255-019-0084-1 PMC7199465

[20] Menk AV , Scharping NE , Rivadeneira DB , Calderon MJ , Watson MJ , Dunstane D , et al. 4‐1BB costimulation induces T cell mitochondrial function and biogenesis enabling cancer immunotherapeutic responses. J Exp Med. 2018;215(4):1091–1100. 29511066 10.1084/jem.20171068 PMC5881463

[21] Tan H , Yang K , Li Y , Shaw TI , Wang Y , Blanco DB , et al. Integrative proteomics and phosphoproteomics profiling reveals dynamic signaling networks and bioenergetics pathways underlying T cell activation. Immunity. 2017;46(3):488–503. 28285833 10.1016/j.immuni.2017.02.010 PMC5466820

[22] Saragovi A , Abramovich I , Omar I , Arbib E , Toker O , Gottlieb E , et al. Systemic hypoxia inhibits T cell response by limiting mitobiogenesis via matrix substrate‐level phosphorylation arrest. elife. 2020;9:e56612. 33226340 10.7554/eLife.56612 PMC7728436

[23] Baixauli F , Martín‐Cófreces NB , Morlino G , Carrasco YR , Calabia‐Linares C , Veiga E , et al. The mitochondrial fission factor dynamin‐related protein 1 modulates T‐cell receptor signalling at the immune synapse. EMBO J. 2011;30(7):1238–1250. 21326213 10.1038/emboj.2011.25 PMC3094108

[24] Quintana A , Pasche M , Junker C , Al‐Ansary D , Rieger H , Kummerow C , et al. Calcium microdomains at the immunological synapse: how ORAI channels, mitochondria and calcium pumps generate local calcium signals for efficient T‐cell activation: calcium microdomains at the immunological synapse. EMBO J. 2011;30(19):3895–3912. 21847095 10.1038/emboj.2011.289 PMC3209779

[25] Sena LA , Li S , Jairaman A , Prakriya M , Ezponda T , Hildeman DA , et al. Mitochondria are required for antigen‐specific T cell activation through reactive oxygen species signaling. Immunity. 2013;38(2):225–236. 23415911 10.1016/j.immuni.2012.10.020 PMC3582741

[26] Hukelmann JL , Anderson KE , Sinclair LV , Grzes KM , Murillo AB , Hawkins PT , et al. The cytotoxic T cell proteome and its shaping by the kinase mTOR. Nat Immunol. 2016;17(1):104–112. 26551880 10.1038/ni.3314 PMC4685757

[27] Tan TCJ , Kelly V , Zou X , Wright D , Ly T , Zamoyska R . Translation factor eIF5a is essential for IFNγ production and cell cycle regulation in primary CD8^+^ T lymphocytes. Nat Commun. 2022;13(1):7796. 36528626 10.1038/s41467-022-35252-y PMC9759561

[28] Marchingo JM , Cantrell DA . Protein synthesis, degradation, and energy metabolism in T cell immunity. Cell Mol Immunol. 2022;19(3):303–315. 34983947 10.1038/s41423-021-00792-8 PMC8891282

[29] Wang R , Dillon CP , Shi LZ , Milasta S , Carter R , Finkelstein D , et al. The transcription factor Myc controls metabolic reprogramming upon T lymphocyte activation. Immunity. 2011;35(6):871–882. 22195744 10.1016/j.immuni.2011.09.021 PMC3248798

[30] Gerriets VA , Kishton RJ , Nichols AG , Macintyre AN , Inoue M , Ilkayeva O , et al. Metabolic programming and PDHK1 control CD4^+^ T cell subsets and inflammation. J Clin Invest. 2015;125(1):194–207. 25437876 10.1172/JCI76012 PMC4382238

[31] Michalek RD , Gerriets VA , Jacobs SR , Macintyre AN , MacIver NJ , Mason EF , et al. Cutting edge: distinct glycolytic and lipid oxidative metabolic programs are essential for effector and regulatory CD4^+^ T cell subsets. J Immunol. 2011;186(6):3299–3303. 21317389 10.4049/jimmunol.1003613 PMC3198034

[32] Chang C‐H , Curtis JD , Maggi LB , Faubert B , Villarino AV , O'Sullivan D , et al. Posttranscriptional control of T cell effector function by aerobic glycolysis. Cell. 2013;153(6):1239–1251. 23746840 10.1016/j.cell.2013.05.016 PMC3804311

[33] Zygmunt BM , Węgrzyn A , Gajska W , Yevsa T , Chodaczek G , Guzmán CA . Mannose metabolism is essential for Th1 cell differentiation and IFN‐γ production. J Immunol. 2018;201(5):1400–1411. 30030325 10.4049/jimmunol.1700042

[34] Metzler B , Gfeller P , Guinet E . Restricting glutamine or glutamine‐dependent purine and pyrimidine syntheses promotes human T cells with high FOXP3 expression and regulatory properties. J Immunol. 2016;196(9):3618–3630. 27022197 10.4049/jimmunol.1501756

[35] Klysz D , Tai X , Robert PA , Craveiro M , Cretenet G , Oburoglu L , et al. Glutamine‐dependent α‐ketoglutarate production regulates the balance between T helper 1 cell and regulatory T cell generation. Sci Signal. 2015;8(396):ra97. 26420908 10.1126/scisignal.aab2610

[36] Matias MI , Yong CS , Foroushani A , Goldsmith C , Mongellaz C , Sezgin E , et al. Regulatory T cell differentiation is controlled by αKG‐induced alterations in mitochondrial metabolism and lipid homeostasis. Cell Rep. 2021;37(5):109911. 34731632 10.1016/j.celrep.2021.109911 PMC10167917

[37] Johnson MO , Wolf MM , Madden MZ , Andrejeva G , Sugiura A , Contreras DC , et al. Distinct regulation of Th17 and Th1 cell differentiation by glutaminase‐dependent metabolism. Cell. 2018;175(7):1780–1795.e19. 30392958 10.1016/j.cell.2018.10.001 PMC6361668

[38] Berod L , Friedrich C , Nandan A , Freitag J , Hagemann S , Harmrolfs K , et al. De novo fatty acid synthesis controls the fate between regulatory T and T helper 17 cells. Nat Med. 2014;20(11):1327–1333. 25282359 10.1038/nm.3704

[39] Angelin A , Gil‐de‐Gómez L , Dahiya S , Jiao J , Guo L , Levine MH , et al. Foxp3 reprograms T cell metabolism to function in low‐glucose, high‐lactate environments. Cell Metab. 2017;25(6):1282–1293.e7. 28416194 10.1016/j.cmet.2016.12.018 PMC5462872

[40] Shi LZ , Wang R , Huang G , Vogel P , Neale G , Green DR , et al. HIF1alpha‐dependent glycolytic pathway orchestrates a metabolic checkpoint for the differentiation of TH17 and Treg cells. J Exp Med. 2011;208(7):1367–1376. 21708926 10.1084/jem.20110278 PMC3135370

[41] Dang EV , Barbi J , Yang H‐Y , Jinasena D , Yu H , Zheng Y , et al. Control of T(H)17/T(reg) balance by hypoxia‐inducible factor 1. Cell. 2011;146(5):772–784. 21871655 10.1016/j.cell.2011.07.033 PMC3387678

[42] Wagner A , Wang C , Fessler J , DeTomaso D , Avila‐Pacheco J , Kaminski J , et al. Metabolic modeling of single Th17 cells reveals regulators of autoimmunity. Cell. 2021;184(16):4168–4185.e21. 34216539 10.1016/j.cell.2021.05.045 PMC8621950

[43] Puleston DJ , Baixauli F , Sanin DE , Edwards‐Hicks J , Villa M , Kabat AM , et al. Polyamine metabolism is a central determinant of helper T cell lineage fidelity. Cell. 2021;184(16):4186–4202.e20. 34216540 10.1016/j.cell.2021.06.007 PMC8358979

[44] Xu T , Stewart KM , Wang X , Liu K , Xie M , Ryu JK , et al. Metabolic control of TH17 and induced Treg cell balance by an epigenetic mechanism. Nature. 2017;548(7666):228–233. 28783731 10.1038/nature23475 PMC6701955

[45] Puleston DJ , Zhang H , Powell TJ , Lipina E , Sims S , Panse I , et al. Autophagy is a critical regulator of memory CD8(+) T cell formation. elife. 2014;3:e03706. 25385531 10.7554/eLife.03706 PMC4225493

[46] Buck MD , O'Sullivan D , Klein Geltink RI , Curtis JD , Chang C‐H , Sanin DE , et al. Mitochondrial dynamics controls T cell fate through metabolic programming. Cell. 2016;166(1):63–76. 27293185 10.1016/j.cell.2016.05.035 PMC4974356

[47] van der Windt GJW , Everts B , Chang C‐H , Curtis JD , Freitas TC , Amiel E , et al. Mitochondrial respiratory capacity is a critical regulator of CD8^+^ T cell memory development. Immunity. 2012;36(1):68–78. 22206904 10.1016/j.immuni.2011.12.007 PMC3269311

[48] Bantug GR , Fischer M , Grählert J , Balmer ML , Unterstab G , Develioglu L , et al. Mitochondria‐endoplasmic reticulum contact sites function as Immunometabolic hubs that orchestrate the rapid recall response of memory CD8^+^ T cells. Immunity. 2018;48(3):542–555.e6. 29523440 10.1016/j.immuni.2018.02.012 PMC6049611

[49] Verbist KC , Guy CS , Milasta S , Liedmann S , Kamiński MM , Wang R , et al. Metabolic maintenance of cell asymmetry following division in activated T lymphocytes. Nature. 2016;532(7599):389–393. 27064903 10.1038/nature17442 PMC4851250

[50] Pollizzi KN , Sun I‐H , Patel CH , Lo Y‐C , Oh M‐H , Waickman AT , et al. Asymmetric inheritance of mTORC1 kinase activity during division dictates CD8^+^ T cell differentiation. Nat Immunol. 2016;17(6):704–711. 27064374 10.1038/ni.3438 PMC4873361

[51] Zheng K , Zheng X , Yang W . The role of metabolic dysfunction in T‐cell exhaustion during chronic viral infection. Front Immunol. 2022;13:843242. 35432304 10.3389/fimmu.2022.843242 PMC9008220

[52] Van Der Heide V , Humblin E , Vaidya A , Kamphorst AO . Advancing beyond the twists and turns of T cell exhaustion in cancer. Sci Transl Med. 2022;14(670):eabo4997. 36350991 10.1126/scitranslmed.abo4997 PMC10000016

[53] Philip M , Schietinger A . CD8^+^ T cell differentiation and dysfunction in cancer. Nat Rev Immunol. 2022;22(4):209–223. 34253904 10.1038/s41577-021-00574-3 PMC9792152

[54] Chang C‐H , Qiu J , O'Sullivan D , Buck MD , Noguchi T , Curtis JD , et al. Metabolic competition in the tumor microenvironment is a driver of cancer progression. Cell. 2015;162(6):1229–1241. 26321679 10.1016/j.cell.2015.08.016 PMC4864363

[55] Patsoukis N , Bardhan K , Chatterjee P , Sari D , Liu B , Bell LN , et al. PD‐1 alters T‐cell metabolic reprogramming by inhibiting glycolysis and promoting lipolysis and fatty acid oxidation. Nat Commun. 2015;6(1):6692. 25809635 10.1038/ncomms7692 PMC4389235

[56] Boussiotis VA , Patsoukis N . Effects of PD‐1 signaling on Immunometabolic reprogramming. Immunometabolism. 2022;4(2):e220007. 35371563 10.20900/immunometab20220007 PMC8975241

[57] Zappasodi R , Serganova I , Cohen IJ , Maeda M , Shindo M , Senbabaoglu Y , et al. CTLA‐4 blockade drives loss of Treg stability in glycolysis‐low tumours. Nature. 2021;591(7851):652–658. 33588426 10.1038/s41586-021-03326-4 PMC8057670

[58] Bengsch B , Johnson AL , Kurachi M , Odorizzi PM , Pauken KE , Attanasio J , et al. Bioenergetic insufficiencies due to metabolic alterations regulated by the inhibitory receptor PD‐1 are an early driver of CD8(^+^) T cell exhaustion. Immunity. 2016;45(2):358–373. 27496729 10.1016/j.immuni.2016.07.008 PMC4988919

[59] Wartewig T , Daniels J , Schulz M , Hameister E , Joshi A , Park J , et al. PD‐1 instructs a tumor‐suppressive metabolic program that restricts glycolysis and restrains AP‐1 activity in T cell lymphoma. Nat Cancer. 2023;4(10):1508–1525. 37723306 10.1038/s43018-023-00635-7 PMC10597841

[60] Kumagai S , Koyama S , Itahashi K , Tanegashima T , Lin Y , Togashi Y , et al. Lactic acid promotes PD‐1 expression in regulatory T cells in highly glycolytic tumor microenvironments. Cancer Cell. 2022;40(2):201–218.e9. 35090594 10.1016/j.ccell.2022.01.001

[61] Scharping NE , Menk AV , Moreci RS , Whetstone RD , Dadey RE , Watkins SC , et al. The tumor microenvironment represses T cell mitochondrial biogenesis to drive Intratumoral T cell metabolic insufficiency and dysfunction. Immunity. 2016;45(2):374–388. 27496732 10.1016/j.immuni.2016.07.009 PMC5207350

[62] Yu Y‐R , Imrichova H , Wang H , Chao T , Xiao Z , Gao M , et al. Disturbed mitochondrial dynamics in CD8^+^ TILs reinforce T cell exhaustion. Nat Immunol. 2020;21(12):1540–1551. 33020660 10.1038/s41590-020-0793-3

[63] Vardhana SA , Hwee MA , Berisa M , Wells DK , Yost KE , King B , et al. Impaired mitochondrial oxidative phosphorylation limits the self‐renewal of T cells exposed to persistent antigen. Nat Immunol. 2020;21(9):1022–1033. 32661364 10.1038/s41590-020-0725-2 PMC7442749

[64] Franco F , Jaccard A , Romero P , Yu Y‐R , Ho P‐C . Metabolic and epigenetic regulation of T‐cell exhaustion. Nat Metab. 2020;2(10):1001–1012. 32958939 10.1038/s42255-020-00280-9

[65] Schurich A , Pallett LJ , Jajbhay D , Wijngaarden J , Otano I , Gill US , et al. Distinct metabolic requirements of exhausted and functional virus‐specific CD8 T cells in the same host. Cell Rep. 2016;16(5):1243–1252. 27452473 10.1016/j.celrep.2016.06.078 PMC4977274

[66] Saxton RA , Knockenhauer KE , Wolfson RL , Chantranupong L , Pacold ME , Wang T , et al. Structural basis for leucine sensing by the Sestrin2‐mTORC1 pathway. Science. 2016;351(6268):53–58. 26586190 10.1126/science.aad2087 PMC4698039

[67] Chantranupong L , Scaria SM , Saxton RA , Gygi MP , Shen K , Wyant GA , et al. The CASTOR proteins are arginine sensors for the mTORC1 pathway. Cell. 2016;165(1):153–164. 26972053 10.1016/j.cell.2016.02.035 PMC4808398

[68] Gu X , Orozco JM , Saxton RA , Condon KJ , Liu GY , Krawczyk PA , et al. SAMTOR is an *S* ‐adenosylmethionine sensor for the mTORC1 pathway. Science. 2017;358(6364):813–818. 29123071 10.1126/science.aao3265 PMC5747364

[69] Sancak Y , Peterson TR , Shaul YD , Lindquist RA , Thoreen CC , Bar‐Peled L , et al. The rag GTPases bind raptor and mediate amino acid signaling to mTORC1. Science. 2008;320(5882):1496–1501. 18497260 10.1126/science.1157535 PMC2475333

[70] Kim SG , Buel GR , Blenis J . Nutrient regulation of the mTOR complex 1 signaling pathway. Mol Cells. 2013;35(6):463–473. 23694989 10.1007/s10059-013-0138-2 PMC3887879

[71] Delgoffe GM , Pollizzi KN , Waickman AT , Heikamp E , Meyers DJ , Horton MR , et al. The kinase mTOR regulates the differentiation of helper T cells through the selective activation of signaling by mTORC1 and mTORC2. Nat Immunol. 2011;12(4):295–303. 21358638 10.1038/ni.2005 PMC3077821

[72] Lee K , Gudapati P , Dragovic S , Spencer C , Joyce S , Killeen N , et al. Mammalian target of rapamycin protein complex 2 regulates differentiation of Th1 and Th2 cell subsets via distinct signaling pathways. Immunity. 2010;32(6):743–753. 20620941 10.1016/j.immuni.2010.06.002 PMC2911434

[73] Delgoffe GM , Kole TP , Zheng Y , Zarek PE , Matthews KL , Xiao B , et al. The mTOR kinase differentially regulates effector and regulatory T cell lineage commitment. Immunity. 2009;30(6):832–844. 19538929 10.1016/j.immuni.2009.04.014 PMC2768135

[74] Sauer S , Bruno L , Hertweck A , Finlay D , Leleu M , Spivakov M , et al. T cell receptor signaling controls Foxp3 expression via PI3K, Akt, and mTOR. Proc Natl Acad Sci USA. 2008;105(22):7797–7802. 18509048 10.1073/pnas.0800928105 PMC2409380

[75] Liu G , Burns S , Huang G , Boyd K , Proia RL , Flavell RA , et al. The receptor S1P1 overrides regulatory T cell‐mediated immune suppression through Akt‐mTOR. Nat Immunol. 2009;10(7):769–777. 19483717 10.1038/ni.1743 PMC2732340

[76] Haxhinasto S , Mathis D , Benoist C . The AKT‐mTOR axis regulates de novo differentiation of CD4^+^Foxp3^+^ cells. J Exp Med. 2008;205(3):565–574. 18283119 10.1084/jem.20071477 PMC2275380

[77] Merkenschlager M , von Boehmer H . PI3 kinase signalling blocks Foxp3 expression by sequestering Foxo factors. J Exp Med. 2010;207(7):1347–1350. 20603315 10.1084/jem.20101156 PMC2901062

[78] Powell JD , Pollizzi KN , Heikamp EB , Horton MR . Regulation of immune responses by mTOR. Annu Rev Immunol. 2012;30:39–68. 22136167 10.1146/annurev-immunol-020711-075024 PMC3616892

[79] Macintyre AN , Gerriets VA , Nichols AG , Michalek RD , Rudolph MC , Deoliveira D , et al. The glucose transporter Glut1 is selectively essential for CD4 T cell activation and effector function. Cell Metab. 2014;20(1):61–72. 24930970 10.1016/j.cmet.2014.05.004 PMC4079750

[80] Sundrud MS , Koralov SB , Feuerer M , Calado DP , Kozhaya AE , Rhule‐Smith A , et al. Halofuginone inhibits TH17 cell differentiation by activating the amino acid starvation response. Science. 2009;324(5932):1334–1338. 19498172 10.1126/science.1172638 PMC2803727

[81] Sinclair LV , Rolf J , Emslie E , Shi Y‐B , Taylor PM , Cantrell DA . Control of amino‐acid transport by antigen receptors coordinates the metabolic reprogramming essential for T cell differentiation. Nat Immunol. 2013;14(5):500–508. 23525088 10.1038/ni.2556 PMC3672957

[82] Nakaya M , Xiao Y , Zhou X , Chang J‐H , Chang M , Cheng X , et al. Inflammatory T cell responses rely on amino acid transporter ASCT2 facilitation of glutamine uptake and mTORC1 kinase activation. Immunity. 2014;40(5):692–705. 24792914 10.1016/j.immuni.2014.04.007 PMC4074507

[83] Shyer JA , Flavell RA , Bailis W . Metabolic signaling in T cells. Cell Res. 2020;30(8):649–659. 32709897 10.1038/s41422-020-0379-5 PMC7395146

[84] Demetriou M , Granovsky M , Quaggin S , Dennis JW . Negative regulation of T‐cell activation and autoimmunity by Mgat5 N‐glycosylation. Nature. 2001;409(6821):733–739. 11217864 10.1038/35055582

[85] Morgan R , Gao G , Pawling J , Dennis JW , Demetriou M , Li B . N‐acetylglucosaminyltransferase V (Mgat5)‐mediated N‐glycosylation negatively regulates Th1 cytokine production by T cells. J Immunol. 2004;173(12):7200–7208. 15585841 10.4049/jimmunol.173.12.7200

[86] Ho P‐C , Bihuniak JD , Macintyre AN , Staron M , Liu X , Amezquita R , et al. Phosphoenolpyruvate is a metabolic checkpoint of anti‐tumor T cell responses. Cell. 2015;162(6):1217–1228. 26321681 10.1016/j.cell.2015.08.012 PMC4567953

[87] Menk AV , Scharping NE , Moreci RS , Zeng X , Guy C , Salvatore S , et al. Early TCR signaling induces rapid aerobic glycolysis enabling distinct acute T cell effector functions. Cell Rep. 2018;22(6):1509–1521. 29425506 10.1016/j.celrep.2018.01.040 PMC5973810

[88] Puleston DJ , Buck MD , Klein Geltink RI , Kyle RL , Caputa G , O'Sullivan D , et al. Polyamines and eIF5A Hypusination modulate mitochondrial respiration and macrophage activation. Cell Metab. 2019;30(2):352–363.e8. 31130465 10.1016/j.cmet.2019.05.003 PMC6688828

[89] Gonzalez‐Menendez P , Phadke I , Olive ME , Joly A , Papoin J , Yan H , et al. Arginine metabolism regulates human erythroid differentiation through hypusination of eIF5A. Blood. 2023;141(20):2520–2536. 36735910 10.1182/blood.2022017584 PMC10273172

[90] Huang Y , Higginson DS , Hester L , Park MH , Snyder SH . Neuronal growth and survival mediated by eIF5A, a polyamine‐modified translation initiation factor. Proc Natl Acad Sci USA. 2007;104(10):4194–4199. 17360499 10.1073/pnas.0611609104 PMC1820731

[91] Soriano‐Baguet L , Grusdat M , Kurniawan H , Benzarti M , Binsfeld C , Ewen A , et al. Pyruvate dehydrogenase fuels a critical citrate pool that is essential for Th17 cell effector functions. Cell Rep. 2023;42(3):112153. 36848289 10.1016/j.celrep.2023.112153

[92] Hochrein SM , Wu H , Eckstein M , Arrigoni L , Herman JS , Schumacher F , et al. The glucose transporter GLUT3 controls T helper 17 cell responses through glycolytic‐epigenetic reprogramming. Cell Metab. 2022;34(4):516–532.e11. 35316657 10.1016/j.cmet.2022.02.015 PMC9019065

[93] Wenes M , Jaccard A , Wyss T , Maldonado‐Pérez N , Teoh ST , Lepez A , et al. The mitochondrial pyruvate carrier regulates memory T cell differentiation and antitumor function. Cell Metab. 2022;34(5):731–746.e9. 35452600 10.1016/j.cmet.2022.03.013 PMC9116152

[94] Gaballa JM , Braga Neto MB , Ramos GP , Bamidele AO , Gonzalez MM , Sagstetter MR , et al. The role of histone methyltransferases and Long non‐coding RNAs in the regulation of T cell fate decisions. Front Immunol. 2018;9:2955. 30619315 10.3389/fimmu.2018.02955 PMC6300512

[95] Dutta A , Venkataganesh H , Love PE . New insights into epigenetic regulation of T cell differentiation. Cells. 2021;10(12):3459. 34943965 10.3390/cells10123459 PMC8700096

[96] Bian Y , Li W , Kremer DM , Sajjakulnukit P , Li S , Crespo J , et al. Cancer SLC43A2 alters T cell methionine metabolism and histone methylation. Nature. 2020;585(7824):277–282. 32879489 10.1038/s41586-020-2682-1 PMC7486248

[97] Jaccard A , Wyss T , Maldonado‐Pérez N , Rath JA , Bevilacqua A , Peng J‐J , et al. Reductive carboxylation epigenetically instructs T cell differentiation. Nature. 2023;621(7980):849–856. 37730993 10.1038/s41586-023-06546-y

[98] Baksh SC , Finley LWS . Metabolic coordination of cell fate by α‐ketoglutarate‐dependent dioxygenases. Trends Cell Biol. 2021;31(1):24–36. 33092942 10.1016/j.tcb.2020.09.010 PMC7748998

[99] Howden AJM , Hukelmann JL , Brenes A , Spinelli L , Sinclair LV , Lamond AI , et al. Quantitative analysis of T cell proteomes and environmental sensors during T cell differentiation. Nat Immunol. 2019;20(11):1542–1554. 31591570 10.1038/s41590-019-0495-x PMC6859072

[100] Wang W , Zou W . Amino acids and their transporters in T cell immunity and cancer therapy. Mol Cell. 2020;80(3):384–395. 32997964 10.1016/j.molcel.2020.09.006 PMC7655528

[101] Kinet S , Swainson L , Lavanya M , Mongellaz C , Montel‐Hagen A , Craveiro M , et al. Isolated receptor binding domains of HTLV‐1 and HTLV‐2 envelopes bind Glut‐1 on activated CD4^+^ and CD8^+^ T cells. Retrovirology. 2007;4:31. 17504522 10.1186/1742-4690-4-31 PMC1876471

[102] Kim FJ , Battini J‐L , Manel N , Sitbon M . Emergence of vertebrate retroviruses and envelope capture. Virology. 2004;318(1):183–191. 14972546 10.1016/j.virol.2003.09.026

[103] Hogan V , Johnson WE . Unique structure and distinctive properties of the ancient and ubiquitous gamma‐type envelope glycoprotein. Viruses. 2023;15(2):274. 36851488 10.3390/v15020274 PMC9967133

[104] Laval J , Touhami J , Herzenberg LA , Conrad C , Taylor N , Battini J‐L , et al. Metabolic adaptation of neutrophils in cystic fibrosis airways involves distinct shifts in nutrient transporter expression. J Immunol. 2013;190(12):6043–6050. 23690474 10.4049/jimmunol.1201755

[105] Klein Geltink RI , Edwards‐Hicks J , Apostolova P , O'Sullivan D , Sanin DE , Patterson AE , et al. Metabolic conditioning of CD8^+^ effector T cells for adoptive cell therapy. Nat Metab. 2020;2(8):703–716. 32747793 10.1038/s42255-020-0256-z PMC10863625

[106] Huang H , Zhou P , Wei J , Long L , Shi H , Dhungana Y , et al. In vivo CRISPR screening reveals nutrient signaling processes underpinning CD8^+^ T cell fate decisions. Cell. 2021;184(5):1245–1261.e21. 33636132 10.1016/j.cell.2021.02.021 PMC8101447

[107] Watson MJ , Vignali PDA , Mullett SJ , Overacre‐Delgoffe AE , Peralta RM , Grebinoski S , et al. Metabolic support of tumour‐infiltrating regulatory T cells by lactic acid. Nature. 2021;591(7851):645–651. 33589820 10.1038/s41586-020-03045-2 PMC7990682

[108] Cretenet G , Clerc I , Matias M , Loisel S , Craveiro M , Oburoglu L , et al. Cell surface Glut1 levels distinguish human CD4 and CD8 T lymphocyte subsets with distinct effector functions. Sci Rep. 2016;6(1):24129. 27067254 10.1038/srep24129 PMC4828702

[109] Arner EN , Rathmell JC . Metabolic programming and immune suppression in the tumor microenvironment. Cancer Cell. 2023;41(3):421–433. 36801000 10.1016/j.ccell.2023.01.009 PMC10023409

[110] Martínez‐Reyes I , Chandel NS . Cancer metabolism: looking forward. Nat Rev Cancer. 2021;21(10):669–680. 34272515 10.1038/s41568-021-00378-6

[111] Van Bruggen JAC , Martens AWJ , Fraietta JA , Hofland T , Tonino SH , Eldering E , et al. Chronic lymphocytic leukemia cells impair mitochondrial fitness in CD8^+^ T cells and impede CAR T‐cell efficacy. Blood. 2019;134(1):44–58. 31076448 10.1182/blood.2018885863 PMC7022375

[112] Siska PJ , Beckermann KE , Mason FM , Andrejeva G , Greenplate AR , Sendor AB , et al. Mitochondrial dysregulation and glycolytic insufficiency functionally impair CD8 T cells infiltrating human renal cell carcinoma. JCI Insight. 2017;2(12):e93411. 28614802 10.1172/jci.insight.93411 PMC5470888

[113] Siska PJ , Van Der Windt GJW , Kishton RJ , Cohen S , Eisner W , MacIver NJ , et al. Suppression of Glut1 and glucose metabolism by decreased Akt/mTORC1 signaling drives T cell impairment in B cell leukemia. J Immunol. 2016;197(6):2532–2540. 27511728 10.4049/jimmunol.1502464 PMC5010978

[114] Reinfeld BI , Madden MZ , Wolf MM , Chytil A , Bader JE , Patterson AR , et al. Cell‐programmed nutrient partitioning in the tumour microenvironment. Nature. 2021;593(7858):282–288. 33828302 10.1038/s41586-021-03442-1 PMC8122068

[115] Gemta LF , Siska PJ , Nelson ME , Gao X , Liu X , Locasale JW , et al. Impaired enolase 1 glycolytic activity restrains effector functions of tumor‐infiltrating CD8^+^ T cells. Sci Immunol. 2019;4(31):eaap9520. 30683669 10.1126/sciimmunol.aap9520 PMC6824424

[116] Lee S‐W , Zhang Y , Jung M , Cruz N , Alas B , Commisso C . EGFR‐Pak signaling selectively regulates glutamine deprivation‐induced macropinocytosis. Dev Cell. 2019;50(3):381–392.e5. 31257175 10.1016/j.devcel.2019.05.043 PMC6684838

[117] Byun J‐K , Park M , Lee S , Yun JW , Lee J , Kim JS , et al. Inhibition of glutamine utilization synergizes with immune checkpoint inhibitor to promote antitumor immunity. Mol Cell. 2020;80(4):592–606.e8. 33159855 10.1016/j.molcel.2020.10.015

[118] Matias MI , Dardalhon V , Taylor N . Targeting glutamine metabolism and PD‐L1: a novel anti‐tumor pas de deux. Mol Cell. 2020;80(4):555–557. 33217313 10.1016/j.molcel.2020.11.005

[119] Pan M , Reid MA , Lowman XH , Kulkarni RP , Tran TQ , Liu X , et al. Regional glutamine deficiency in tumours promotes dedifferentiation through inhibition of histone demethylation. Nat Cell Biol. 2016;18(10):1090–1101. 27617932 10.1038/ncb3410 PMC5536113

[120] Geiger R , Rieckmann JC , Wolf T , Basso C , Feng Y , Fuhrer T , et al. L‐arginine modulates T cell metabolism and enhances survival and anti‐tumor activity. Cell. 2016;167(3):829–842.e13. 27745970 10.1016/j.cell.2016.09.031 PMC5075284

[121] West EE , Merle NS , Kamiński MM , Palacios G , Kumar D , Wang L , et al. Loss of CD4^+^ T cell‐intrinsic arginase 1 accelerates Th1 response kinetics and reduces lung pathology during influenza infection. Immunity. 2023;56(9):2036–2053.e12. 37572656 10.1016/j.immuni.2023.07.014 PMC10576612

[122] Wu J , Li G , Li L , Li D , Dong Z , Jiang P . Asparagine enhances LCK signalling to potentiate CD8^+^ T‐cell activation and anti‐tumour responses. Nat Cell Biol. 2021;23(1):75–86. 33420490 10.1038/s41556-020-00615-4

[123] Wei J , Raynor J , Nguyen T‐LM , Chi H . Nutrient and metabolic sensing in T cell responses. Front Immunol. 2017;8:247. 28337199 10.3389/fimmu.2017.00247 PMC5343023

[124] Ma EH , Bantug G , Griss T , Condotta S , Johnson RM , Samborska B , et al. Serine is an essential metabolite for effector T cell expansion. Cell Metab. 2017;25(2):345–357. 28111214 10.1016/j.cmet.2016.12.011

[125] Edwards DN , Ngwa VM , Raybuck AL , Wang S , Hwang Y , Kim LC , et al. Selective glutamine metabolism inhibition in tumor cells improves antitumor T lymphocyte activity in triple‐negative breast cancer. J Clin Invest. 2021;131(4):e140100. 33320840 10.1172/JCI140100 PMC7880417

[126] Grzywa TM , Sosnowska A , Matryba P , Rydzynska Z , Jasinski M , Nowis D , et al. Myeloid cell‐derived arginase in cancer immune response. Front Immunol. 2020;11:938. 32499785 10.3389/fimmu.2020.00938 PMC7242730

[127] Czystowska‐Kuzmicz M , Sosnowska A , Nowis D , Ramji K , Szajnik M , Chlebowska‐Tuz J , et al. Small extracellular vesicles containing arginase‐1 suppress T‐cell responses and promote tumor growth in ovarian carcinoma. Nat Commun. 2019;10(1):3000. 31278254 10.1038/s41467-019-10979-3 PMC6611910

[128] Ino Y , Yamazaki‐Itoh R , Oguro S , Shimada K , Kosuge T , Zavada J , et al. Arginase II expressed in cancer‐associated fibroblasts indicates tissue hypoxia and predicts poor outcome in patients with pancreatic cancer. PLoS One. 2013;8(2):e55146. 23424623 10.1371/journal.pone.0055146 PMC3570471

[129] Bron L , Jandus C , Andrejevic‐Blant S , Speiser DE , Monnier P , Romero P , et al. Prognostic value of arginase‐II expression and regulatory T‐cell infiltration in head and neck squamous cell carcinoma. Int J Cancer. 2013;132(3):E85–E93. 22815199 10.1002/ijc.27728

[130] Brand A , Singer K , Koehl GE , Kolitzus M , Schoenhammer G , Thiel A , et al. LDHA‐associated lactic acid production blunts tumor immunosurveillance by T and NK cells. Cell Metab. 2016;24(5):657–671. 27641098 10.1016/j.cmet.2016.08.011

[131] Hermans D , Gautam S , García‐Cañaveras JC , Gromer D , Mitra S , Spolski R , et al. Lactate dehydrogenase inhibition synergizes with IL‐21 to promote CD8^+^ T cell stemness and antitumor immunity. Proc Natl Acad Sci USA. 2020;117(11):6047–6055. 32123114 10.1073/pnas.1920413117 PMC7084161

[132] Feng Q , Liu Z , Yu X , Huang T , Chen J , Wang J , et al. Lactate increases stemness of CD8^+^ T cells to augment anti‐tumor immunity. Nat Commun. 2022;13(1):4981. 36068198 10.1038/s41467-022-32521-8 PMC9448806

[133] Terness P , Bauer TM , Röse L , Dufter C , Watzlik A , Simon H , et al. Inhibition of allogeneic T cell proliferation by indoleamine 2,3‐dioxygenase–expressing dendritic cells. J Exp Med. 2002;196(4):447–457. 12186837 10.1084/jem.20020052 PMC2196057

[134] Uyttenhove C , Pilotte L , Théate I , Stroobant V , Colau D , Parmentier N , et al. Evidence for a tumoral immune resistance mechanism based on tryptophan degradation by indoleamine 2,3‐dioxygenase. Nat Med. 2003;9(10):1269–1274. 14502282 10.1038/nm934

[135] Campesato LF , Budhu S , Tchaicha J , Weng C‐H , Gigoux M , Cohen IJ , et al. Blockade of the AHR restricts a Treg‐macrophage suppressive axis induced by L‐kynurenine. Nat Commun. 2020;11(1):4011. 32782249 10.1038/s41467-020-17750-z PMC7419300

[136] Liu Y , Liang X , Yin X , Lv J , Tang K , Ma J , et al. Blockade of IDO‐kynurenine‐AhR metabolic circuitry abrogates IFN‐γ‐induced immunologic dormancy of tumor‐repopulating cells. Nat Commun. 2017;8(1):15207. 28488695 10.1038/ncomms15207 PMC5436221

[137] Liu Y , Liang X , Dong W , Fang Y , Lv J , Zhang T , et al. Tumor‐repopulating cells induce PD‐1 expression in CD8^+^ T cells by transferring kynurenine and AhR activation. Cancer Cell. 2018;33(3):480–494.e7. 29533786 10.1016/j.ccell.2018.02.005

[138] The Multiple Leiomyoma Consortium . Germline mutations in FH predispose to dominantly inherited uterine fibroids, skin leiomyomata and papillary renal cell cancer. Nat Genet. 2002;30(4):406–410. 11865300 10.1038/ng849

[139] Valcarcel‐Jimenez L , Frezza C . Fumarate hydratase (FH) and cancer: a paradigm of oncometabolism. Br J Cancer. 2023;129(10):1546–1557. 37689804 10.1038/s41416-023-02412-w PMC10645937

[140] Cheng J , Yan J , Liu Y , Shi J , Wang H , Zhou H , et al. Cancer‐cell‐derived fumarate suppresses the anti‐tumor capacity of CD8^+^ T cells in the tumor microenvironment. Cell Metab. 2023;35(6):961–978.e10. 37178684 10.1016/j.cmet.2023.04.017

[141] Notarangelo G , Spinelli JB , Perez EM , Baker GJ , Kurmi K , Elia I , et al. Oncometabolite d‐2HG alters T cell metabolism to impair CD8^+^ T cell function. Science. 2022;377(6614):1519–1529. 36173860 10.1126/science.abj5104 PMC9629749

[142] Foskolou IP , Cunha PP , Sánchez‐López E , Minogue EA , Nicolet BP , Guislain A , et al. The two enantiomers of 2‐hydroxyglutarate differentially regulate cytotoxic T cell function. Cell Rep. 2023;42(9):113013. 37632752 10.1016/j.celrep.2023.113013 PMC7618115

[143] Eil R , Vodnala SK , Clever D , Klebanoff CA , Sukumar M , Pan JH , et al. Ionic immune suppression within the tumour microenvironment limits T cell effector function. Nature. 2016;537(7621):539–543. 27626381 10.1038/nature19364 PMC5204372

[144] Ong ST , Ng AS , Ng XR , Zhuang Z , Wong BHS , Prasannan P , et al. Extracellular K^+^ dampens T cell functions: implications for immune suppression in the tumor microenvironment. Bioelectricity. 2019;1(3):169–179. 34471819 10.1089/bioe.2019.0016 PMC8370284

[145] Vodnala SK , Eil R , Kishton RJ , Sukumar M , Yamamoto TN , Ha N‐H , et al. T cell stemness and dysfunction in tumors are triggered by a common mechanism. Science. 2019;363(6434):eaau0135. 30923193 10.1126/science.aau0135 PMC8194369

[146] Zhao H , Teng D , Yang L , Xu X , Chen J , Jiang T , et al. Myeloid‐derived itaconate suppresses cytotoxic CD8^+^ T cells and promotes tumour growth. Nat Metab. 2022;4(12):1660–1673. 36376563 10.1038/s42255-022-00676-9 PMC10593361

[147] Di Virgilio F , Sarti AC , Falzoni S , De Marchi E , Adinolfi E . Extracellular ATP and P2 purinergic signalling in the tumour microenvironment. Nat Rev Cancer. 2018;18(10):601–618. 30006588 10.1038/s41568-018-0037-0

[148] Cekic C , Linden J . Purinergic regulation of the immune system. Nat Rev Immunol. 2016;16(3):177–192. 26922909 10.1038/nri.2016.4

[149] Yegutkin GG , Henttinen T , Samburski SS , Spychala J , Jalkanen S . The evidence for two opposite, ATP‐generating and ATP‐consuming, extracellular pathways on endothelial and lymphoid cells. Biochem J. 2002;367(1):121–128. 12099890 10.1042/BJ20020439 PMC1222875

[150] Ohta A , Sitkovsky M . Role of G‐protein‐coupled adenosine receptors in downregulation of inflammation and protection from tissue damage. Nature. 2001;414(6866):916–920. 11780065 10.1038/414916a

[151] Krishna S , Lowery FJ , Copeland AR , Bahadiroglu E , Mukherjee R , Jia L , et al. Stem‐like CD8 T cells mediate response of adoptive cell immunotherapy against human cancer. Science. 2020;370(6522):1328–1334. 33303615 10.1126/science.abb9847 PMC8883579

[152] Vignali PDA , DePeaux K , Watson MJ , Ye C , Ford BR , Lontos K , et al. Hypoxia drives CD39‐dependent suppressor function in exhausted T cells to limit antitumor immunity. Nat Immunol. 2023;24(2):267–279. 36543958 10.1038/s41590-022-01379-9 PMC10402660

[153] Najjar YG , Menk AV , Sander C , Rao U , Karunamurthy A , Bhatia R , et al. Tumor cell oxidative metabolism as a barrier to PD‐1 blockade immunotherapy in melanoma. JCI Insight. 2019;4(5):e124989. 30721155 10.1172/jci.insight.124989 PMC6483505

[154] Scharping NE , Rivadeneira DB , Menk AV , Vignali PDA , Ford BR , Rittenhouse NL , et al. Mitochondrial stress induced by continuous stimulation under hypoxia rapidly drives T cell exhaustion. Nat Immunol. 2021;22(2):205–215. 33398183 10.1038/s41590-020-00834-9 PMC7971090

[155] Chimento A , Casaburi I , Avena P , Trotta F , De Luca A , Rago V , et al. Cholesterol and its metabolites in tumor growth: therapeutic potential of statins in cancer treatment. Front Endocrinol. 2019;9:807. 10.3389/fendo.2018.00807 PMC6348274 30719023

[156] Ma X , Bi E , Lu Y , Su P , Huang C , Liu L , et al. Cholesterol induces CD8^+^ T cell exhaustion in the tumor microenvironment. Cell Metab. 2019;30(1):143–156.e5. 31031094 10.1016/j.cmet.2019.04.002 PMC7061417

[157] Yang W , Bai Y , Xiong Y , Zhang J , Chen S , Zheng X , et al. Potentiating the antitumour response of CD8^+^ T cells by modulating cholesterol metabolism. Nature. 2016;531(7596):651–655. 26982734 10.1038/nature17412 PMC4851431

[158] Ma X , Bi E , Huang C , Lu Y , Xue G , Guo X , et al. Cholesterol negatively regulates IL‐9–producing CD8^+^ T cell differentiation and antitumor activity. J Exp Med. 2018;215(6):1555–1569. 29743292 10.1084/jem.20171576 PMC5987919

[159] Jin H‐R , Wang J , Wang Z‐J , Xi M‐J , Xia B‐H , Deng K , et al. Lipid metabolic reprogramming in tumor microenvironment: from mechanisms to therapeutics. J Hematol Oncol. 2023;16(1):103. 37700339 10.1186/s13045-023-01498-2 PMC10498649

[160] Chen JH , Perry CJ , Tsui Y‐C , Staron MM , Parish IA , Dominguez CX , et al. Prostaglandin E2 and programmed cell death 1 signaling coordinately impair CTL function and survival during chronic viral infection. Nat Med. 2015;21(4):327–334. 25799228 10.1038/nm.3831 PMC4505619

[161] Chemnitz JM , Driesen J , Classen S , Riley JL , Debey S , Beyer M , et al. Prostaglandin E2 impairs CD4^+^ T cell activation by inhibition of lck: implications in Hodgkin's lymphoma. Cancer Res. 2006;66(2):1114–1122. 16424048 10.1158/0008-5472.CAN-05-3252

[162] Baratelli F , Lin Y , Zhu L , Yang S‐C , Heuzé‐Vourc'h N , Zeng G , et al. Prostaglandin E2 induces *FOXP3* gene expression and T regulatory cell function in human CD4^+^ T cells. J Immunol. 2005;175(3):1483–1490. 16034085 10.4049/jimmunol.175.3.1483

[163] Bayerl F , Meiser P , Donakonda S , Hirschberger A , Lacher SB , Pedde A‐M , et al. Tumor‐derived prostaglandin E2 programs cDC1 dysfunction to impair intratumoral orchestration of anti‐cancer T cell responses. Immunity. 2023;56(6):1341–1358.e11. 37315536 10.1016/j.immuni.2023.05.011

[164] Maude SL , Frey N , Shaw PA , Aplenc R , Barrett DM , Bunin NJ , et al. Chimeric antigen receptor T cells for sustained remissions in leukemia. N Engl J Med. 2014;371(16):1507–1517. 25317870 10.1056/NEJMoa1407222 PMC4267531

[165] Lee DW , Kochenderfer JN , Stetler‐Stevenson M , Cui YK , Delbrook C , Feldman SA , et al. T cells expressing CD19 chimeric antigen receptors for acute lymphoblastic leukaemia in children and young adults: a phase 1 dose‐escalation trial. Lancet. 2015;385(9967):517–528. 25319501 10.1016/S0140-6736(14)61403-3 PMC7065359

[166] Park JH , Rivière I , Gonen M , Wang X , Sénéchal B , Curran KJ , et al. Long‐term follow‐up of CD19 CAR therapy in acute lymphoblastic leukemia. N Engl J Med. 2018;378(5):449–459. 29385376 10.1056/NEJMoa1709919 PMC6637939

[167] Shah NN , Fry TJ . Mechanisms of resistance to CAR T cell therapy. Nat Rev Clin Oncol. 2019;16:372–385. 30837712 10.1038/s41571-019-0184-6 PMC8214555

[168] Knochelmann HM , Smith AS , Dwyer CJ , Wyatt MM , Mehrotra S , Paulos CM . CAR T cells in solid tumors: blueprints for building effective therapies. Front Immunol. 2018;9:1740. 30140266 10.3389/fimmu.2018.01740 PMC6094980

[169] Lugli E , Dominguez MH , Gattinoni L , Chattopadhyay PK , Bolton DL , Song K , et al. Superior T memory stem cell persistence supports long‐lived T cell memory. J Clin Invest. 2013;JCI66327. 10.1172/JCI66327 PMC3561805 23281401

[170] Klebanoff CA , Scott CD , Leonardi AJ , Yamamoto TN , Cruz AC , Ouyang C , et al. Memory T cell–driven differentiation of naive cells impairs adoptive immunotherapy. J Clin Invest. 2015;126(1):318–334. 26657860 10.1172/JCI81217 PMC4701537

[171] Sabatino M , Hu J , Sommariva M , Gautam S , Fellowes V , Hocker JD , et al. Generation of clinical‐grade CD19‐specific CAR‐modified CD8^+^ memory stem cells for the treatment of human B‐cell malignancies. Blood. 2016;128(4):519–528. 27226436 10.1182/blood-2015-11-683847 PMC4965906

[172] Fraietta JA , Lacey SF , Orlando EJ , Pruteanu‐Malinici I , Gohil M , Lundh S , et al. Determinants of response and resistance to CD19 chimeric antigen receptor (CAR) T cell therapy of chronic lymphocytic leukemia. Nat Med. 2018;24(5):563–571. 29713085 10.1038/s41591-018-0010-1 PMC6117613

[173] Sukumar M , Liu J , Ji Y , Subramanian M , Crompton JG , Yu Z , et al. Inhibiting glycolytic metabolism enhances CD8^+^ T cell memory and antitumor function. J Clin Invest. 2013;123(10):4479–4488. 24091329 10.1172/JCI69589 PMC3784544

[174] Hong M , Talluri S , Chen YY . Advances in promoting chimeric antigen receptor T cell trafficking and infiltration of solid tumors. Curr Opin Biotechnol. 2023;84:103020. 37976958 10.1016/j.copbio.2023.103020

[175] Beavis PA , Slaney CY , Kershaw MH , Gyorki D , Neeson PJ , Darcy PK . Reprogramming the tumor microenvironment to enhance adoptive cellular therapy. Semin Immunol. 2016;28(1):64–72. 26611350 10.1016/j.smim.2015.11.003

[176] Albelda SM . CAR T cell therapy for patients with solid tumours: key lessons to learn and unlearn. Nat Rev Clin Oncol. 2024;21(1):47–66. 37904019 10.1038/s41571-023-00832-4

[177] Humblin E , Kamphorst AO . CXCR3‐CXCL9: It's all in the tumor. Immunity. 2019;50(6):1347–1349. 31216458 10.1016/j.immuni.2019.05.013

[178] Chow MT , Ozga AJ , Servis RL , Frederick DT , Lo JA , Fisher DE , et al. Intratumoral activity of the CXCR3 chemokine system is required for the efficacy of anti‐PD‐1 therapy. Immunity. 2019;50(6):1498–1512.e5. 31097342 10.1016/j.immuni.2019.04.010 PMC6527362

[179] Xu Y , Zhang M , Ramos CA , Durett A , Liu E , Dakhova O , et al. Closely related T‐memory stem cells correlate with in vivo expansion of CAR.CD19‐T cells and are preserved by IL‐7 and IL‐15. Blood. 2014;123(24):3750–3759. 24782509 10.1182/blood-2014-01-552174 PMC4055922

[180] Zhou J , Jin L , Wang F , Zhang Y , Liu B , Zhao T . Chimeric antigen receptor T (CAR‐T) cells expanded with IL‐7/IL‐15 mediate superior antitumor effects. Protein Cell. 2019;10(10):764–769. 31250350 10.1007/s13238-019-0643-y PMC6776495

[181] Battram AM , Bachiller M , Lopez V , Fernández de Larrea C , Urbano‐Ispizua A , Martín‐Antonio B . IL‐15 enhances the persistence and function of BCMA‐targeting CAR‐T cells compared to IL‐2 or IL‐15/IL‐7 by limiting CAR‐T cell dysfunction and differentiation. Cancer. 2021;13(14):3534. 10.3390/cancers13143534 PMC8304527 34298748

[182] Alizadeh D , Wong RA , Yang X , Wang D , Pecoraro JR , Kuo C‐F , et al. IL15 enhances CAR‐T cell antitumor activity by reducing mTORC1 activity and preserving their stem cell memory phenotype. Cancer Immunol Res. 2019;7(5):759–772. 30890531 10.1158/2326-6066.CIR-18-0466 PMC6687561

[183] Alvarez‐Fernández C , Escribà‐Garcia L , Vidal S , Sierra J , Briones J . A short CD3/CD28 costimulation combined with IL‐21 enhance the generation of human memory stem T cells for adoptive immunotherapy. J Transl Med. 2016;14(1):214. 27435312 10.1186/s12967-016-0973-y PMC4952071

[184] Jeza VT , Li X , Chen J , Liang Z , Aggrey AO , Wu X . IL‐21 augments rapamycin in expansion of alpha fetoprotein antigen specific stem‐cell‐like memory T cells in vitro. Pan Afr Med J. 2017;27:163. 28904691 10.11604/pamj.2017.27.163.11072 PMC5567945

[185] Loschinski R , Böttcher M , Stoll A , Bruns H , Mackensen A , Mougiakakos D . IL‐21 modulates memory and exhaustion phenotype of T‐cells in a fatty acid oxidation‐dependent manner. Oncotarget. 2018;9(17):13125–13138. 29568345 10.18632/oncotarget.24442 PMC5862566

[186] Ghassemi S , Durgin JS , Nunez‐Cruz S , Patel J , Leferovich J , Pinzone M , et al. Rapid manufacturing of non‐activated potent CAR T cells. Nat Biomed Eng. 2022;6(2):118–128. 35190680 10.1038/s41551-021-00842-6 PMC8860360

[187] Mo F , Yu Z , Li P , Oh J , Spolski R , Zhao L , et al. An engineered IL‐2 partial agonist promotes CD8^+^ T cell stemness. Nature. 2021;597(7877):544–548. 34526724 10.1038/s41586-021-03861-0 PMC9172917

[188] Funk CR , Wang S , Chen KZ , Waller A , Sharma A , Edgar CL , et al. PI3Kδ/γ inhibition promotes human CART cell epigenetic and metabolic reprogramming to enhance antitumor cytotoxicity. Blood. 2022;139(4):523–537. 35084470 10.1182/blood.2021011597 PMC8796652

[189] Stock S , Übelhart R , Schubert M , Fan F , He B , Hoffmann J , et al. Idelalisib for optimized CD19‐specific chimeric antigen receptor T cells in chronic lymphocytic leukemia patients. Int J Cancer. 2019;145(5):1312–1324. 30737788 10.1002/ijc.32201

[190] Zheng W , O'Hear CE , Alli R , Basham JH , Abdelsamed HA , Palmer LE , et al. PI3K orchestration of the in vivo persistence of chimeric antigen receptor‐modified T cells. Leukemia. 2018;32(5):1157–1167. 29479065 10.1038/s41375-017-0008-6 PMC5943191

[191] Crompton JG , Sukumar M , Roychoudhuri R , Clever D , Gros A , Eil RL , et al. Akt inhibition enhances expansion of potent tumor‐specific lymphocytes with memory cell characteristics. Cancer Res. 2015;75(2):296–305. 25432172 10.1158/0008-5472.CAN-14-2277 PMC4384335

[192] Nian Z , Zheng X , Dou Y , Du X , Zhou L , Fu B , et al. Rapamycin pretreatment rescues the bone marrow AML cell elimination capacity of CAR‐T cells. Clin Cancer Res. 2021;27(21):6026–6038. 34233960 10.1158/1078-0432.CCR-21-0452 PMC9401534

[193] Mousset CM , Hobo W , Ji Y , Fredrix H , De Giorgi V , Allison RD , et al. *Ex vivo* AKT‐inhibition facilitates generation of polyfunctional stem cell memory‐like CD8^+^ T cells for adoptive immunotherapy. Onco Targets Ther. 2018;7(10):e1488565. 10.1080/2162402X.2018.1488565 PMC6169586 30288356

[194] Klebanoff CA , Crompton JG , Leonardi AJ , Yamamoto TN , Chandran SS , Eil RL , et al. Inhibition of AKT signaling uncouples T cell differentiation from expansion for receptor‐engineered adoptive immunotherapy. JCI Insight. 2017;2(23):e95103. 29212954 10.1172/jci.insight.95103 PMC5752304

[195] Gattinoni L , Zhong X‐S , Palmer DC , Ji Y , Hinrichs CS , Yu Z , et al. Wnt signaling arrests effector T cell differentiation and generates CD8^+^ memory stem cells. Nat Med. 2009;15(7):808–813. 19525962 10.1038/nm.1982 PMC2707501

[196] Gattinoni L , Lugli E , Ji Y , Pos Z , Paulos CM , Quigley MF , et al. A human memory T cell subset with stem cell–like properties. Nat Med. 2011;17(10):1290–1297. 21926977 10.1038/nm.2446 PMC3192229

[197] Kondo T , Morita R , Okuzono Y , Nakatsukasa H , Sekiya T , Chikuma S , et al. Notch‐mediated conversion of activated T cells into stem cell memory‐like T cells for adoptive immunotherapy. Nat Commun. 2017;8(1):15338. 28530241 10.1038/ncomms15338 PMC5458121

[198] Kondo T , Ando M , Nagai N , Tomisato W , Srirat T , Liu B , et al. The NOTCH–FOXM1 Axis plays a key role in mitochondrial biogenesis in the induction of human stem cell memory–like CAR‐T cells. Cancer Res. 2020;80(3):471–483. 31767627 10.1158/0008-5472.CAN-19-1196

[199] Ando M , Kondo T , Tomisato W , Ito M , Shichino S , Srirat T , et al. Rejuvenating effector/exhausted CAR T cells to stem cell memory–like CAR T cells by resting them in the presence of CXCL12 and the NOTCH ligand. Cancer Res Commun. 2021;1(1):41–55. 36860911 10.1158/2767-9764.CRC-21-0034 PMC9973402

[200] Denk D , Petrocelli V , Conche C , Drachsler M , Ziegler PK , Braun A , et al. Expansion of T memory stem cells with superior anti‐tumor immunity by urolithin A‐induced mitophagy. Immunity. 2022;55(11):2059–2073.e8. 36351375 10.1016/j.immuni.2022.09.014

[201] Marchesi F , Vignali D , Manini B , Rigamonti A , Monti P . Manipulation of glucose availability to boost cancer immunotherapies. Cancer. 2020;12(10):2940. 10.3390/cancers12102940 PMC7650629 33053779

[202] Lopez E , Karattil R , Nannini F , Weng‐Kit Cheung G , Denzler L , Galvez‐Cancino F , et al. Inhibition of lactate transport by MCT‐1 blockade improves chimeric antigen receptor T‐cell therapy against B‐cell malignancies. J Immunother Cancer. 2023;11(6):e006287. 37399358 10.1136/jitc-2022-006287 PMC10314680

[203] Lv Z , Guo Y . Metformin and its benefits for various diseases. Front Endocrinol. 2020;11:191. 10.3389/fendo.2020.00191 PMC7212476 32425881

[204] Zhang Z , Li F , Tian Y , Cao L , Gao Q , Zhang C , et al. Metformin enhances the antitumor activity of CD8^+^ T lymphocytes via the AMPK–miR‐107–Eomes–PD‐1 pathway. J Immunol. 2020;204(9):2575–2588. 32221038 10.4049/jimmunol.1901213

[205] Chao Y , Wei T , Li Q , Liu B , Hao Y , Chen M , et al. Metformin‐containing hydrogel scaffold to augment CAR‐T therapy against post‐surgical solid tumors. Biomaterials. 2023;295:122052. 36827893 10.1016/j.biomaterials.2023.122052

[206] Lontos K , Wang Y , Joshi SK , Frisch AT , Watson MJ , Kumar A , et al. Metabolic reprogramming via an engineered PGC‐1α improves human chimeric antigen receptor T‐cell therapy against solid tumors. J Immunother Cancer. 2023;11(3):e006522. 36914208 10.1136/jitc-2022-006522 PMC10016249

[207] Zhao Z , Condomines M , van der Stegen SJC , Perna F , Kloss CC , Gunset G , et al. Structural design of engineered costimulation determines tumor rejection kinetics and persistence of CAR T cells. Cancer Cell. 2015;28(4):415–428. 26461090 10.1016/j.ccell.2015.09.004 PMC5003056

[208] Kowolik CM , Topp MS , Gonzalez S , Pfeiffer T , Olivares S , Gonzalez N , et al. CD28 costimulation provided through a CD19‐specific chimeric antigen receptor enhances *in vivo* persistence and antitumor efficacy of adoptively transferred T cells. Cancer Res. 2006;66(22):10995–11004. 17108138 10.1158/0008-5472.CAN-06-0160

[209] Philipson BI , O'Connor RS , May MJ , June CH , Albelda SM , Milone MC . 4‐1BB costimulation promotes CAR T cell survival through noncanonical NF‐κB signaling. Sci Signal. 2020;13(625):eaay8248. 32234960 10.1126/scisignal.aay8248 PMC7883633

[210] Li G , Boucher JC , Kotani H , Park K , Zhang Y , Shrestha B , et al. 4‐1BB enhancement of CAR T function requires NF‐κB and TRAFs. JCI Insight. 2018;3(18):e121322. 30232281 10.1172/jci.insight.121322 PMC6237232

[211] Long AH , Haso WM , Shern JF , Wanhainen KM , Murgai M , Ingaramo M , et al. 4‐1BB costimulation ameliorates T cell exhaustion induced by tonic signaling of chimeric antigen receptors. Nat Med. 2015;21(6):581–590. 25939063 10.1038/nm.3838 PMC4458184

[212] Kawalekar OU , O'Connor RS , Fraietta JA , Guo L , McGettigan SE , Posey AD , et al. Distinct signaling of coreceptors regulates specific metabolism pathways and impacts memory development in CAR T cells. Immunity. 2016;44(2):380–390. 26885860 10.1016/j.immuni.2016.01.021 PMC13498396

[213] Fultang L , Booth S , Yogev O , Martins Da Costa B , Tubb V , Panetti S , et al. Metabolic engineering against the arginine microenvironment enhances CAR‐T cell proliferation and therapeutic activity. Blood. 2020;136(10):1155–1160. 32573723 10.1182/blood.2019004500 PMC7565134

[214] Panetti S , McJannett N , Fultang L , Booth S , Gneo L , Scarpa U , et al. Engineering amino acid uptake or catabolism promotes CAR T‐cell adaption to the tumor environment. Blood Adv. 2023;7(9):1754–1761. 36521029 10.1182/bloodadvances.2022008272 PMC10182289

[215] McCutcheon SR , Swartz AM , Brown MC , Barrera A , McRoberts Amador C , Siklenka K , et al. Transcriptional and epigenetic regulators of human CD8^+^ T cell function identified through orthogonal CRISPR screens. Nat Genet. 2023;55:2211–2223. 10.1038/s41588-023-01554-0 37945901 PMC10703699

[216] Wei J , Long L , Zheng W , Dhungana Y , Lim SA , Guy C , et al. Targeting REGNASE‐1 programs long‐lived effector T cells for cancer therapy. Nature. 2019;576(7787):471–476. 31827283 10.1038/s41586-019-1821-z PMC6937596

[217] Ye L , Park JJ , Peng L , Yang Q , Chow RD , Dong MB , et al. A genome‐scale gain‐of‐function CRISPR screen in CD8 T cells identifies proline metabolism as a means to enhance CAR‐T therapy. Cell Metab. 2022;34(4):595–614.e14. 35276062 10.1016/j.cmet.2022.02.009 PMC8986623

[218] Shifrut E , Carnevale J , Tobin V , Roth TL , Woo JM , Bui CT , et al. Genome‐wide CRISPR screens in primary human T cells reveal key regulators of immune function. Cell. 2018;175(7):1958–1971.e15. 30449619 10.1016/j.cell.2018.10.024 PMC6689405

[219] Wang D , Prager BC , Gimple RC , Aguilar B , Alizadeh D , Tang H , et al. CRISPR screening of CAR T cells and cancer stem cells reveals critical dependencies for cell‐based therapies. Cancer Discov. 2021;11(5):1192–1211. 33328215 10.1158/2159-8290.CD-20-1243 PMC8406797

[220] Shang W , Jiang Y , Boettcher M , Ding K , Mollenauer M , Liu Z , et al. Genome‐wide CRISPR screen identifies FAM49B as a key regulator of actin dynamics and T cell activation. Proc Natl Acad Sci USA. 2018;115(17):E4051–E4060. 29632189 10.1073/pnas.1801340115 PMC5924929

[221] Xin G , Schauder DM , Lainez B , Weinstein JS , Dai Z , Chen Y , et al. A critical role of IL‐21‐induced BATF in sustaining CD8‐T‐cell‐mediated chronic viral control. Cell Rep. 2015;13(6):1118–1124. 26527008 10.1016/j.celrep.2015.09.069 PMC4859432

[222] Seo H , González‐Avalos E , Zhang W , Ramchandani P , Yang C , Lio C‐WJ , et al. BATF and IRF4 cooperate to counter exhaustion in tumor‐infiltrating CAR T cells. Nat Immunol. 2021;22(8):983–995. 34282330 10.1038/s41590-021-00964-8 PMC8319109

[223] Ataide MA , Komander K , Knöpper K , Peters AE , Wu H , Eickhoff S , et al. BATF3 programs CD8^+^ T cell memory. Nat Immunol. 2020;21(11):1397–1407. 32989328 10.1038/s41590-020-0786-2

[224] Zhang X , Zhang C , Qiao M , Cheng C , Tang N , Lu S , et al. Depletion of BATF in CAR‐T cells enhances antitumor activity by inducing resistance against exhaustion and formation of central memory cells. Cancer Cell. 2022;40(11):1407–1422.e7. 36240777 10.1016/j.ccell.2022.09.013

[225] Jain N , Zhao Z , Feucht J , Koche R , Iyer A , Dobrin A , et al. TET2 guards against unchecked BATF3‐induced CAR T cell expansion. Nature. 2023;615(7951):315–322. 36755094 10.1038/s41586-022-05692-z PMC10511001

[226] Yang J , Chen Y , Jing Y , Green MR , Han L . Advancing CAR T cell therapy through the use of multidimensional omics data. Nat Rev Clin Oncol. 2023;20(4):211–228. 36721024 10.1038/s41571-023-00729-2 PMC11734589

[227] Hartmann FJ , Mrdjen D , McCaffrey E , Glass DR , Greenwald NF , Bharadwaj A , et al. Single‐cell metabolic profiling of human cytotoxic T cells. Nat Biotechnol. 2021;39(2):186–197. 32868913 10.1038/s41587-020-0651-8 PMC7878201

[228] Argüello RJ , Combes AJ , Char R , Gigan J‐P , Baaziz AI , Bousiquot E , et al. SCENITH: a flow cytometry‐based method to functionally profile energy metabolism with single‐cell resolution. Cell Metab. 2020;32(6):1063–1075.e7. 33264598 10.1016/j.cmet.2020.11.007 PMC8407169

[229] Hu T , Allam M , Cai S , Henderson W , Yueh B , Garipcan A , et al. Single‐cell spatial metabolomics with cell‐type specific protein profiling for tissue systems biology. Nat Commun. 2023;14(1):8260. 38086839 10.1038/s41467-023-43917-5 PMC10716522

[230] Xiao Z , Dai Z , Locasale JW . Metabolic landscape of the tumor microenvironment at single cell resolution. Nat Commun. 2019;10(1):3763. 31434891 10.1038/s41467-019-11738-0 PMC6704063

[231] Heintzman DR , Fisher EL , Rathmell JC . Microenvironmental influences on T cell immunity in cancer and inflammation. Cell Mol Immunol. 2022;19(3):316–326. 35039633 10.1038/s41423-021-00833-2 PMC8762638

[232] Hung MH , Lee JS , Ma C , Diggs LP , Heinrich S , Chang CW , et al. Tumor methionine metabolism drives T‐cell exhaustion in hepatocellular carcinoma. Nat Commun. 2021;12(1):1455. 33674593 10.1038/s41467-021-21804-1 PMC7935900

[233] Manzo T , Prentice BM , Anderson KG , Raman A , Schalck A , Codreanu GS , et al. Accumulation of long‐chain fatty acids in the tumor microenvironment drives dysfunction in intrapancreatic CD8^+^ T cells. J Exp Med. 2020;217(8):e20191920. 32491160 10.1084/jem.20191920 PMC7398173

[234] Sun Y , Wu L , Zhong Y , Zhou K , Hou Y , Wang Z , et al. Single‐cell landscape of the ecosystem in early‐relapse hepatocellular carcinoma. Cell. 2021;184(2):404–421.e16. 33357445 10.1016/j.cell.2020.11.041

[235] Bao J , Yu Y . Identification of a prognostic evaluator from glutamine metabolic heterogeneity studies within and between tissues in hepatocellular carcinoma. Front Pharmacol. 2023;14:1241677. 37954858 10.3389/fphar.2023.1241677 PMC10637396

[236] Liu Y , Beyer A , Aebersold R . On the dependency of cellular protein levels on mRNA abundance. Cell. 2016;165(3):535–550. 27104977 10.1016/j.cell.2016.03.014

[237] Purohit V , Wagner A , Yosef N , Kuchroo VK . Systems‐based approaches to study immunometabolism. Cell Mol Immunol. 2022;19(3):409–420. 35121805 10.1038/s41423-021-00783-9 PMC8891302

[238] Mestas J , Hughes CCW . Of mice and not men: differences between mouse and human immunology. J Immunol. 2004;172(5):2731–2738. 14978070 10.4049/jimmunol.172.5.2731

[239] Liszewski MK , Kolev M , Le Friec G , Leung M , Bertram PG , Fara AF , et al. Intracellular complement activation sustains T cell homeostasis and mediates effector differentiation. Immunity. 2013;39(6):1143–1157. 24315997 10.1016/j.immuni.2013.10.018 PMC3865363

[240] Freeley S , Kemper C , Le Friec G . The “ins and outs” of complement‐driven immune responses. Immunol Rev. 2016;274(1):16–32. 27782335 10.1111/imr.12472 PMC5102160

[241] Rosshart SP , Vassallo BG , Angeletti D , Hutchinson DS , Morgan AP , Takeda K , et al. Wild mouse gut microbiota promotes host fitness and improves disease resistance. Cell. 2017;171(5):1015–1028.e13. 29056339 10.1016/j.cell.2017.09.016 PMC6887100

[242] Rosshart SP , Herz J , Vassallo BG , Hunter A , Wall MK , Badger JH , et al. Laboratory mice born to wild mice have natural microbiota and model human immune responses. Science. 2019;365(6452):eaaw4361. 31371577 10.1126/science.aaw4361 PMC7377314

[243] Sallusto F . Host response: mice and humans in the bubble. Nat Microbiol. 2016;1(7):16105. 27572983 10.1038/nmicrobiol.2016.105

[244] Beura LK , Hamilton SE , Bi K , Schenkel JM , Odumade OA , Casey KA , et al. Normalizing the environment recapitulates adult human immune traits in laboratory mice. Nature. 2016;532(7600):512–516. 27096360 10.1038/nature17655 PMC4871315

[245] Reese TA , Bi K , Kambal A , Filali‐Mouhim A , Beura LK , Bürger MC , et al. Sequential infection with common pathogens promotes human‐like immune gene expression and altered vaccine response. Cell Host Microbe. 2016;19(5):713–719. 27107939 10.1016/j.chom.2016.04.003 PMC4896745

[246] Bygrave AE , Rose KL , Cortes‐Hernandez J , Warren J , Rigby RJ , Cook HT , et al. Spontaneous autoimmunity in 129 and C57BL/6 mice—implications for autoimmunity described in gene‐targeted mice. PLoS Biol. 2004;2(8):E243. 15314659 10.1371/journal.pbio.0020243 PMC509305

[247] Morel L . Mouse models of human autoimmune diseases: essential tools that require the proper controls. PLoS Biol. 2004;2(8):E241. 15314657 10.1371/journal.pbio.0020241 PMC509303

[248] Hofmann SM , Landgraf R . Research in metabolic ageing — a tale of mice versus humans? Nat Rev Endocrinol. 2022;18(1):7–8. 34824384 10.1038/s41574-021-00597-9

[249] Palliyaguru DL , Shiroma EJ , Nam JK , Duregon E , Vieira Ligo Teixeira C , Price NL , et al. Fasting blood glucose as a predictor of mortality: lost in translation. Cell Metab. 2021;33(11):2189–2200.e3. 34508697 10.1016/j.cmet.2021.08.013 PMC9115768

